# Supplementary material for: Epithelial cells maintain memory of prior infection with Streptococcus pneumoniae through di-methylation of histone H3
Source: Nat Commun. 2024 Jul 2;15:5545. doi: 10.1038/s41467-024-49347-1 (PMC11219877; doi:10.1038/s41467-024-49347-1)
Supplement: Supplementary file 1 — Supplementary Information [file 41467_2024_49347_MOESM1_ESM.pdf]

Figure S1

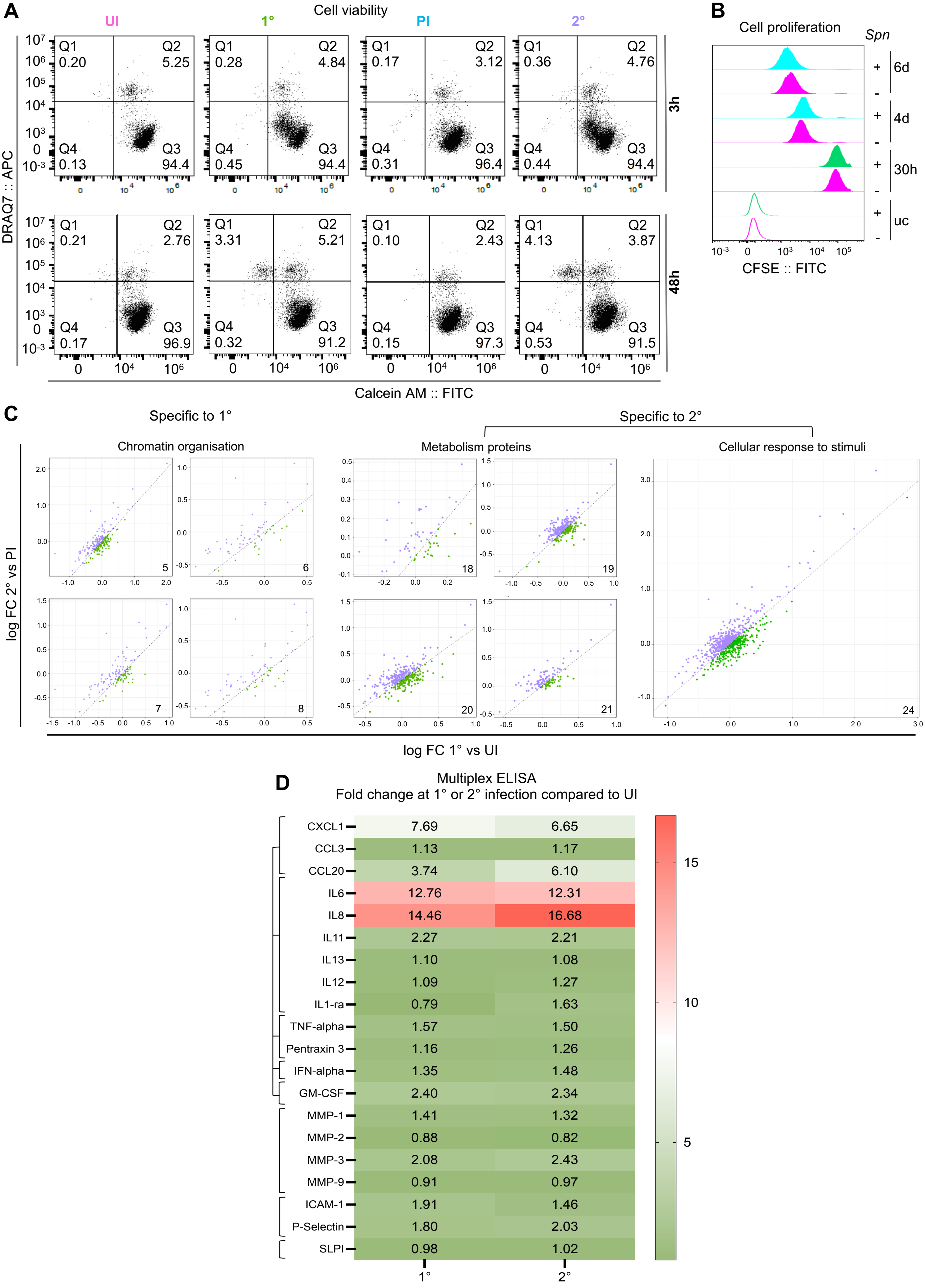

A
Cell viability
UI
1°
PI
2°
3h
48h
DRAQ7 :: APC
Calcein AM :: FITC
Q1 0.20 Q2 5.25 Q4 0.13 Q3 94.4
Q1 0.28 Q2 4.84 Q4 0.45 Q3 94.4
Q1 0.17 Q2 3.12 Q4 0.31 Q3 96.4
Q1 0.36 Q2 4.76 Q4 0.44 Q3 94.4
Q1 0.21 Q2 2.76 Q4 0.17 Q3 96.9
Q1 3.31 Q2 5.21 Q4 0.32 Q3 91.2
Q1 0.10 Q2 2.43 Q4 0.15 Q3 97.3
Q1 4.13 Q2 3.87 Q4 0.53 Q3 91.5
B
Cell proliferation
Spn
6d
4d
30h
uc
CFSE :: FITC
C
Specific to 1°
Specific to 2°
Chromatin organisation
Metabolism proteins
Cellular response to stimuli
log FC 2° vs PI
log FC 1° vs UI
D
Multiplex ELISA
Fold change at 1° or 2° infection compared to UI
CXCL1 7.69 6.65
CCL3 1.13 1.17
CCL20 3.74 6.10
IL6 12.76 12.31
IL8 14.46 16.68
IL11 2.27 2.21
IL13 1.10 1.08
IL12 1.09 1.27
IL1-ra 0.79 1.63
TNF-alpha 1.57 1.50
Pentraxin 3 1.16 1.26
IFN-alpha 1.35 1.48
GM-CSF 2.40 2.34
MMP-1 1.41 1.32
MMP-2 0.88 0.82
MMP-3 2.08 2.43
MMP-9 0.91 0.97
ICAM-1 1.91 1.46
P-Selectin 1.80 2.03
SLPI 0.98 1.02
1°
2°
15
10
5

Figure S1: **Differential infection efficiency and host cell transcriptome between primary and secondary infections**

(A) Cell viability analysis. A549 cells uninfected or infected (MOI 20) collected at time α (3h) and γ (48h), traited with Calcein AM (living cell dye) and DRAQ7^{TM} (death cell dye) then analyzed by flow cytometry. Quatrant gates split the population into 4 subpopulations (Q1-4)): double positive population Calcein AM/DRAQ7^{TM} (Q2), double negative population Calcein AM/DRAQ7^{TM} (Q4), single positive cell Calcein AM (Q3) and single positive cell DRAQ7^{TM} (Q1). The gate frequencies displayed are the percentage of events failing within each Q, with respect to the events that fall in the analyzed population. **(B)** Cell proliferation analysis. Every generation of A549 cells stained with CFSE appears as a different full peak and unstained (uc) appears as no-full peaks on flow cytometry histogram for UI (-) and infected with *Spn* (+) cells at 30 hours (30h), 4 days (4d), 6 days (6d) post infection and unstained control (uc). **(C)** Comparison of expression changes between infections for significantly enriched pathways. Plots show the log fold change between for the primary infection (1° vs UI) in comparison to the secondary infection (2° vs PI) for the genes of the enriched pathways of the Chromatin organisation, Metabolism proteins and Cellular response to stimuli REACTOME categories. **(D)** Inflammatory cytokines analysis by Multiplex ELISA. Quantification of twenty cytokines simultaneously at time α (3h) from supernatant of uninfected and infected A549 cells (MOI 20, at 1°, 2° and PI). Table heatmaps representing fold change of mean at 1° to UI and 2° to PI. Table display cytokines quantification from n=7 biological replicates with the mean values (pg/mL) of each condition. Source data are provided as a Source Data file for A-D.

Table S1: Reactome pathways and corresponding categories

Reactome category significantly enriched in primary infection	No / Pathways	1° *NES	1° **FDR <0.05	2° *NES	2° **FDR >0.05
Disease (Infection)					
	1 Export of viral ribonucleoproteins from nucleus	-2.03	0.0013	-1.34	0.4211
	2 HCMV early events	-1.88	0.0003	-1.05	0.6668
Cellular responses to stimuli					
	3 DNA damage telomere stress induced senescence	-2.07	0.0002	-1.44	0.2190
	4 Cellular senescence	-1.69	0.0030	1.42	0.1113
Chromatin organization					
	5 Chromatin modifying enzymes	-1.75	0.0001	-1.29	0.2003
	6 PKMTS methylate histone lysines	-2.26	2.28e-05	-1.39	0.3097
	7 HATS acetylate histones	-1.93	0.0001	-1.22	0.4435
	8 RMTS methylate histone arginines	-1.93	0.0020	-1.18	0.5358
Gene Expression					
	9 Nuclear receptor transcription pathway	2.24	8.62e-05	1.64	0.1307
Signal transduction					
	10 GPCR (G-protein coupled receptor) ligand binding	1.55	0.0020	1.36	0.1137
	11 Class A1 rhodopsin like receptors (largest group of GPCRs)	1.72	0.0006	1.3	0.2223
	12 Peptide ligand binding receptors (subset of the Class A/1 family, peptide ligands which include the chemokines)	1.90	0.0002	1.34	0.1881
	13 Chemokine receptors bind chemokines	2.33	3.01e-05	1.54	0.2190

Reactome category significantly enriched in secondary infection	No / Pathways	1° *NES	1° **FDR >0.05	2° *NES	2° **FDR <0.05
Signal transduction					
	14 Signaling by nuclear receptors	1.21	0.2829	1.60	0.0048
	15 Negative regulation of MAPK pathway	1.65	0.0663	2.07	0.0048
Metabolism proteins					
	16 Activation of the mRNA upon binding of the cap binding complex and EIFS and subsequent binding to 43S	1.17	0.5814	2.29	5.47e-05
	17 Translation	0.8	1	1.85	7.15e-06
	18 Metabolism of amino acids and derivatives	1.07	0.5994	1.80	3.41e-05
	19 Eukaryotic translation initiation	1.54	0.0521	2.63	5.429e-11
Disease (infection)					
	20 Infectious disease	0.98	0.8427	1.54	5.47e-05
	21 Influenza infection	1.22	0.3767	2.1	6.89e-06
Cellular responses to stimuli					
	22 Cellular response to external stimuli	-1.07	0.5782	1.72	3.94e-07

Reactome category common to both infection	No / Pathways	1° *NES	1° **FDR <0.05	2° *NES	2° **FDR <0.05
Signal transduction					
	23 Rho GTPase cycle	-1.61	0.0002	-1.53	0.0022
	24 Signaling by Rho GTPases miro GTPases and RhoBTB3	-1.84	1.91e-10	-1.47	0.0010
Programmed cell death					
	25 Apoptotic execution phase	-1.97	0.0027	-1.92	0.0078
	26 Apoptosis induced DNA fragmentation	-2.1	0.0007	-2.02	0.0011
DNA repair					
	27 DNA repair	-2.27	2.21e-13	-1.76	3.51e-05
	28 HDR through homologous recombination (HRR)	-2.18	8.63e-05	-1.78	0.0228
	29 Homology directed repair (HDR)	-2.32	5.33e-08	-1.66	0.0228
	30 Homologous DNA pairing and strand exchange	-2.34	1.72e-05	-1.88	0.0134
	31 Resolution of Dloop structures through synthesis dependent strand annealing (SDSA)	-2.21	0.0002	-2.04	0.0075
	32 DNA double strand break repair	-2.28	9.02e-09	-1.66	0.0056
	33 Resolution of D loop structures	-2.17	0.0002	-2.05	0.0022
Gene Expression					
	34 RNA polymerase II transcription	-1.95	2.72e-21	-1.67	8.18e-11
	35 FOXO mediated transcription	2.03	0.0006	2.14	0.0003
Cellular responses to stimuli					
	36 Response of EIF2AK4 GCN2 to amino acid deficiency	1.87	0.0013	2.74	6.73e-12
	37 Cellular response to starvation	1.55	0.0344	2.32	3.22e-08
Immune system					
	38 Signaling by Interleukins	1.85	1.05e-06	1.83	4.62e-06
	39 Interleukin 4 and Interleukin 13 signaling	2.30	1.01e-06	2.21	2.21e-05
	40 Interleukin 10 signaling	2.20	0.0005	2.32	0.0003
	41 Cytokine signaling in immune system	1.60	6.65e-05	1.54	0.0003
	42 Toll like receptor TLR1 TLR2 cascade	1.66	0.0160	1.88	0.0040

*NES: Normalized enrichment score **FDR: False discovery rate

Figure S2

A

		UI	1°	PI	2°
3h	Cells n=	63	53	54	62
	Lysosomes n=	8438	8588	6171	8769
24h	Cells n=	67	74	68	71
	Lysosomes n=	21729	18406	19208	110023

B

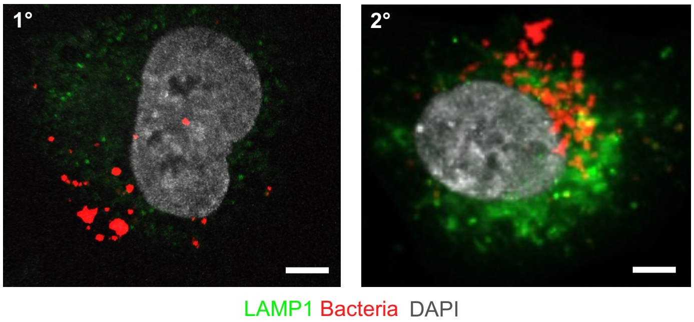

C

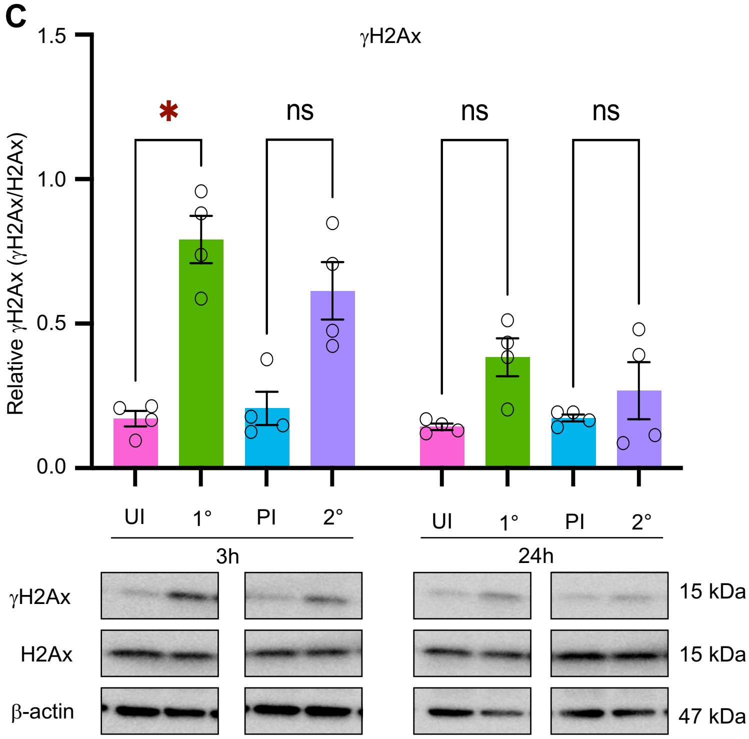

Figure S2: *S. pneumoniae* actively modifies cells following primary infection

(A) Quantity of A549 cells and lysosomes analyzed for the quantification of LAMP1 and colocalization with lysotracker® for UI, 1°, PI and 2° conditions at time α and β. See Figure 2.D. (**B**) Representative images of immunofluorescence confocal microscopy of A549 cells stained LAMP1 (GFP; green), bacteria (WGA488; red) and nucleus (DAPI; grey) at time α (3h) after 1°and 2° infection. Crop of images taken at 63x magnification. Scale bar = 5 µm. **(C)** Immunoblot detection of γH2Ax from infected (1°, 2°, PI, with MOI 20) and uninfected A549 cells at time α and β. Histogram show actin-normalized ratio of γH2Ax to total H2Ax from n=4 biological replicates. Mean ± SEM of γH2Ax levels. Statistical significance was determined by two-way ANOVA comparing all means with Tukey's multiple comparisons test (ns = not significant, *p =0.0333). See bellow representative image of immunoblot detection of γH2Ax for each condition. Source data are provided as a Source Data file for A-C.

Figure S3

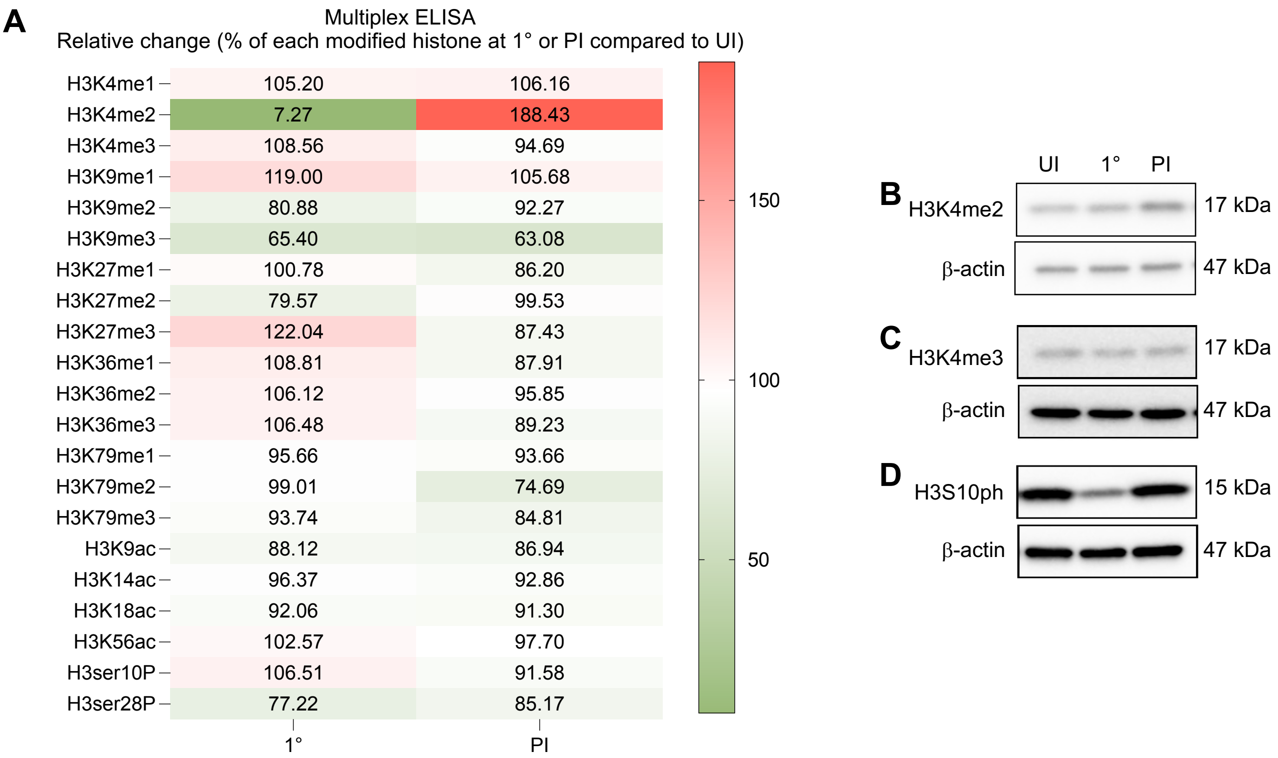

A
Multiplex ELISA
Relative change (% of each modified histone at 1° or PI compared to UI)
H3K4me1 105.20 106.16
H3K4me2 7.27 188.43
H3K4me3 108.56 94.69
H3K9me1 119.00 105.68
H3K9me2 80.88 92.27
H3K9me3 65.40 63.08
H3K27me1 100.78 86.20
H3K27me2 79.57 99.53
H3K27me3 122.04 87.43
H3K36me1 108.81 87.91
H3K36me2 106.12 95.85
H3K36me3 106.48 89.23
H3K79me1 95.66 93.66
H3K79me2 99.01 74.69
H3K79me3 93.74 84.81
H3K9ac 88.12 86.94
H3K14ac 96.37 92.86
H3K18ac 92.06 91.30
H3K56ac 102.57 97.70
H3ser10P 106.51 91.58
H3ser28P 77.22 85.17
1°
PI
150
100
50
UI
1°
PI
B
H3K4me2
17 kDa
β-actin
47 kDa
C
H3K4me3
17 kDa
β-actin
47 kDa
D
H3S10ph
15 kDa
β-actin
47 kDa

Figure S3: H3K4me2 is a specific mark lasting beyond primary infection

(A) Quantification of twenty-one modified histone H3 patterns by Multiplex ELISA assay in the A549 cells at 1° and PI at time α (3h). Table heatmaps representing total H3-normalized ratio of the relative change (%) of each histone H3 modification between 1° or PI and UI. **(B-D)** Representative image of immunoblot detection of H3K4me2 **(B)**, H3K4me3 **(C)**, H3S10ph **(D)** and β-actin from lysed UI and infected (1° and PI) A549 cells at time α. See quantification Figures 3B, C, D. Source data are provided as a Source Data file for A-D.

Figure S4

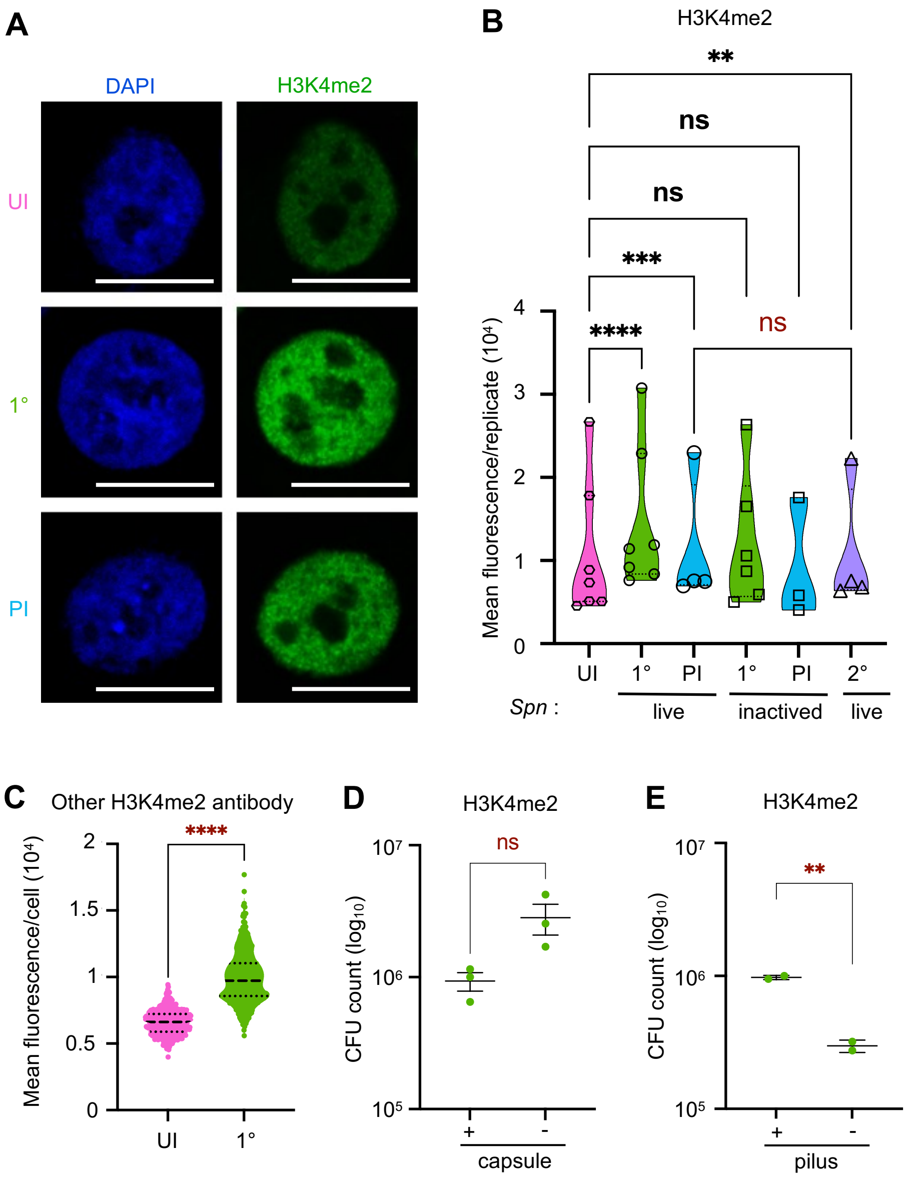
A
DAPI
H3K4me2
UI
1°
PI
B
H3K4me2
**
ns
ns
***
****
ns
Mean fluorescence/replicate (10^4)
0
1
2
3
4
UI
1°
PI
1°
PI
2°
Spn :
live
inactived
live
C
Other H3K4me2 antibody
****
Mean fluorescence/cell (10^4)
0
0.5
1
1.5
2
UI
1°
D
H3K4me2
ns
CFU count ($\log_{10}$)
10^7
10^6
10^5
+
-
capsule
E
H3K4me2
**
CFU count ($\log_{10}$)
10^7
10^6
10^5
+
-
pilus

Figure S4: The increase in H3K4me2 levels is actively induced by live bacteria and dependent on cellular binding

(A). Representative images of immunofluorescence confocal microscopy detection of H3K4me2 in nuclear of A549 cells UI and infected (1° and PI) at time γ. Cells stained for H3K4me2 (GFP; green) and nuclei (DAPI; blue). Crop of Images taken at 63x magnification. Scale bar is 10μm. **(B)** Quantification of H3K4me2 normalized to the segmented nuclei using DAPI signal. Data points expressed mean fluorescence intensity for each biological replicates (n= 4-7) for UI, infected A549 cells (1° and PI with MOI 20) with *sp* live and *sp* inactived, infected cells (2°, MOI 20) with *Spn* live, at time γ. Violin plot (truncated) show all points, statistical significance was determined by ANOVA with matching across each biological replicat and Tukey's multiple comparaisons test with a single pooled variance (ns = not significant, **p = 0.005, ***p <0.0009, ****p <0.0001). **(C)** Quantification of nuclear H3K4me2 from RPMI 2650 cells normalized to the segmented nuclei using DAPI signal Data points represent the mean fluorescence intensity (MFI) of H3K4me2 (Epigentek antibody) within individual nuclei. Violin plot (truncated) show n=500 nuclei at 1° MOI 10 and UI at time γ, statistical significance was determined by Mann-Whitney test (****p <0.0001). **(D)** A549 cells were collected after 1° (MOI 10) at time α (3h) with *Spn* mutant without capsule (-) compared *Spn* wildtype (+) for CFU counts, n=3 biological replicates, lines are the mean ± SEM and statistical significance was calculated by unpaired t test, ns = not significant. (**E**) A549 cells were collected at time α (3h) after 1° (MOI 10) with *Spn* mutant without pilus (-) compared *Spn* wildtype (+) for CFU counts, n=2 biological replicates, lines are the mean ± SEM and statistical significance was calculated by unpaired t test, **p = 0.0025. Source data are provided as a Source Data file for A-E.

Figure S5

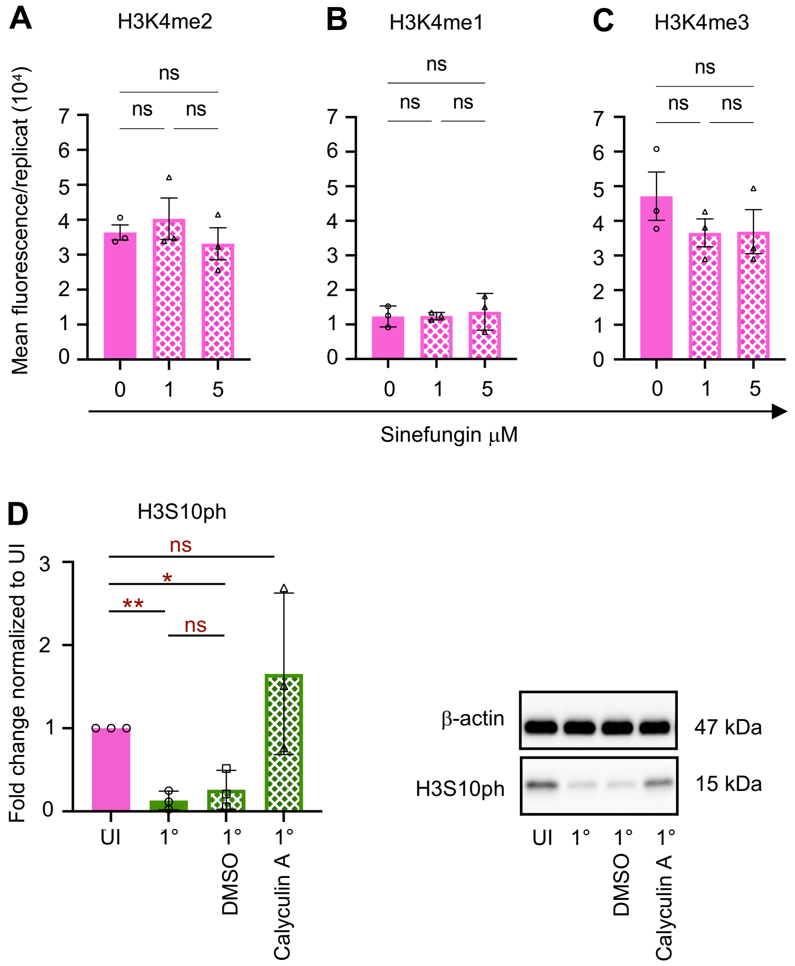

A
H3K4me2
B
H3K4me1
C
H3K4me3
ns
Mean fluorescence/replicat (10⁴)
0
1
5
Sinefungin μM
D
H3S10ph
ns
*
**
Fold change normalized to UI
UI
1°
DMSO
Calyculin A
β-actin
H3S10ph
47 kDa
15 kDa

Figure S5: H3K4me2 is a specific persistent mark induced by infection

(A-B-C) Quantification of H3K4me2 **(A)**, H3K4me1 **(B)**, H3K4me3 **(C)** normalized to the segmented nuclei using DAPI signal. Data points expressed as mean fluorescence intensity of UI for each biological replicat (n=3) ± methyltransferase global inhibitor Sinefungin (2 differents doses, μM) at time γ (3h) for H3K4me2 and H3K4me1 and at time β (24h) for H3K4me3 (See experimental set-up Figure 5.C). Statistical significance was determined by one-way ANOVA comparing means with Tukey's multiple comparison test (ns = not significant). See traited and infected cells in Figure 5D, E, F. **(D)** Immunoblot detection of H3S10ph from UI and infected (1°, MOI 20) whole A549 cell lysates ± traited with DMSO or 0.05 μM Calyculin A at time α (3h) (see experimental set-up Figure 5G). Histogram show mean ± SEM of values expressed as normalized band intensity relative to β-actin followed by fold change of infected cells at 1° to UI for n=3 biological replicates. Statistical significance was determined by two-tailed paired t test (ns = not significant, *p =0.0319, **p =0.0055). On the right, representative image of immunoblot detection of H3S10ph and β-actin for each condition. Source data are provided as a Source Data file for A-D.

Figure S6

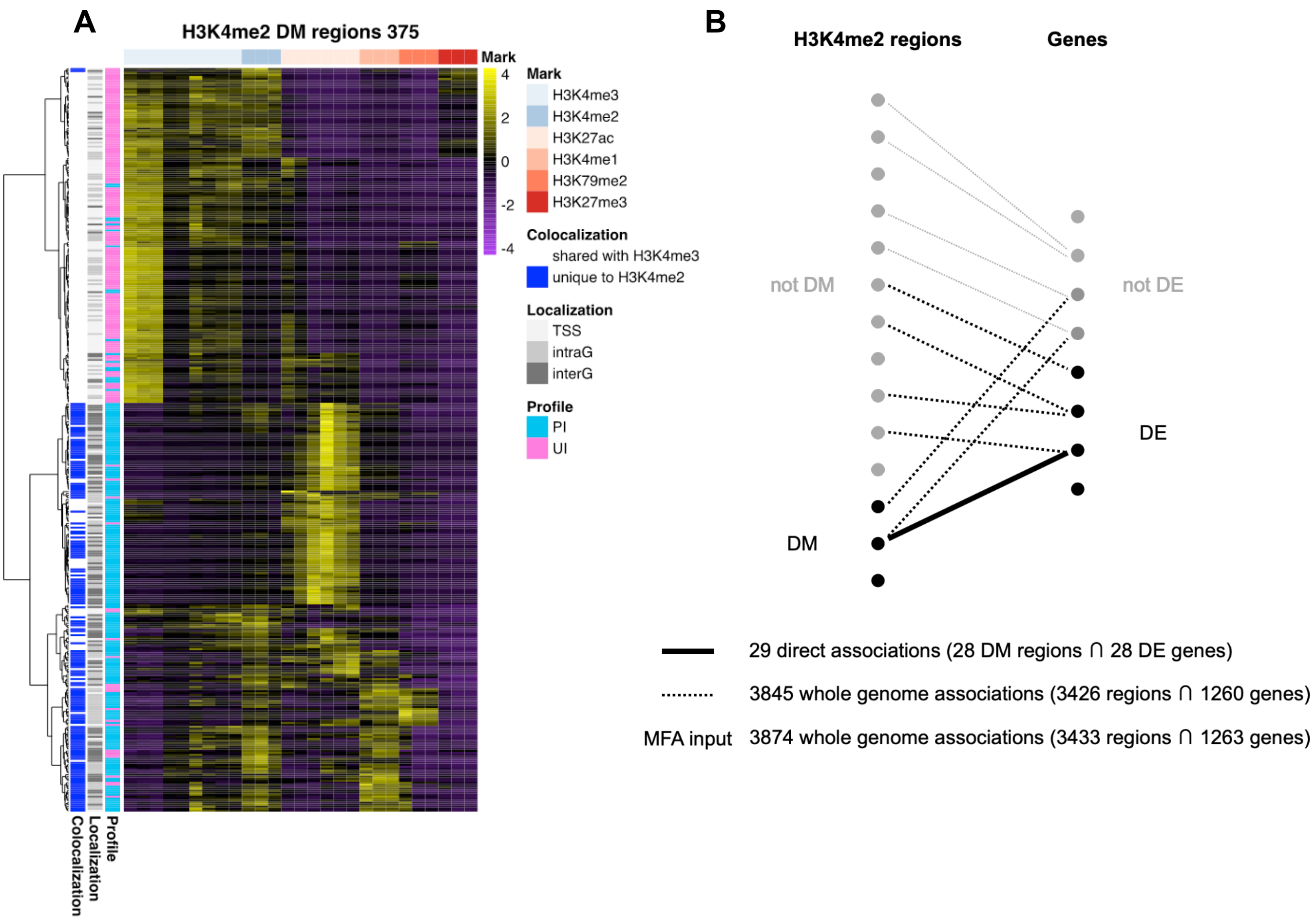

A
H3K4me2 DM regions 375
Mark
Mark
H3K4me3
H3K4me2
H3K27ac
H3K4me1
H3K79me2
H3K27me3
Colocalization
shared with H3K4me3
unique to H3K4me2
Localization
TSS
intraG
interG
Profile
PI
UI
4
2
0
-2
-4
Colocalization
Localization
Profile
B
H3K4me2 regions
Genes
not DM
not DE
DE
DM
29 direct associations (28 DM regions ∩ 28 DE genes)
3845 whole genome associations (3426 regions ∩ 1260 genes)
MFA input 3874 whole genome associations (3433 regions ∩ 1263 genes)

Figure S6: Chromatin profiling over H3K4me2 differentially methylated peaks and regulatory association to genes

(A) Chromatin profiling over the H3K4me2 dynamic peaks. Coverage for key histone modifications recovered from the ENCODE portal for A549 cells is shown for the 375 H3K4me2 dynamic peaks. Histone coverage is normalised by peak length, centered and scaled among samples. Peaks are clustered according to their chromatin state and annotated according to their profile: PI (gain of methylation) or UI (loss of methylation); their localization with respect to the nearest gene (TSS = overlapping the 2 Kb interval centred around the transcription start site; IntraG = located within the gene annotations and outside the TSS interval; InterG = all other peaks); the colocalization with H3K4me3 dynamic peaks (shared or unique). **(B)** Association between methylome and transcriptome. Dots represent H3K4me2 regions or genes and lines are regulatory links predicted by the T-Gene tool of the MEME suite. Black lines (continuous and dotted) show regulatory links where either a DMR and/or a DEG are involved. These account for the 3874 (29 + 3845) associations used as input for the multiple factor analysis (MFA). Grey lines represent regulatory links between not DMs and not DEs and are therefore not included in the downstream integrative analysis. Source Data A-B were deposited into the Gene Expression Omnibus (GEO) repository of the National Center for Biotechnology Information under accession number GSE230142.

Figure S7

A

Quantitative variables by group

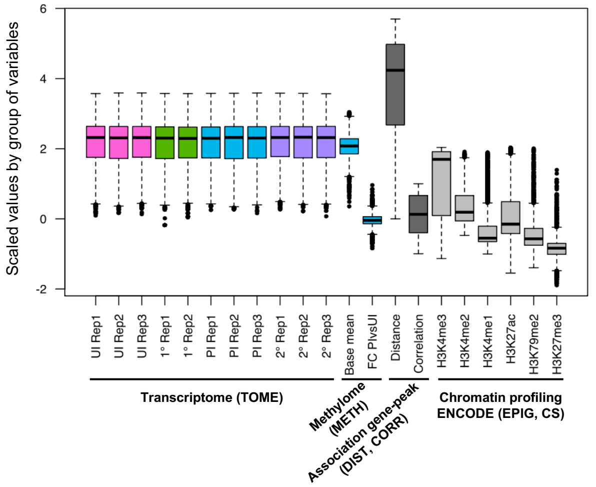

Categorical variables by group

Transcriptome

DEG kind \ DEG sign	Up	Down	Up-Down	Down-Up
**Only 1°**	55	109	172	165
**Only 2°**	41	31	102	104
**Not specific**	161	179	70	74

Methylome

DMR kind \ DMR sign	Gain	Loss
**DM**	111	154
**Not DM**	1282	1886

B

Dimension / Factor	Cumulative % of variance
**Dim 1**	15,8
**Dim 2**	28,8
**Dim 3**	39,2
**Dim 4**	48,1
**Dim 5**	56,5

Group of variables	Dim 1	Dim 2	Dim 3	Dim 4	Dim 5
**TOME**	0,28	0,28	0,64	0,10	0,06
**METH**	0,30	0,54	0,46	0,77	0,39
**DIST**	0,07	0,52	0,24	0,28	0,57
**CORR**	0,00	0,21	0,46	0,50	0,20
**EPIG**	0,12	0,76	0,41	0,29	0,22
**DEG**	0,95	0,15	0,47	0,25	0,54
**DMR**	0,95	0,67	0,29	0,16	0,52

C

**MFA cluster 1**	p.value	v.test
DM sign=Gain	0,0E+00	26,14
DM kind=notDM	0,0E+00	10,86
CS=6	0,0E+00	10,39
CS=3	0,0E+00	7,99
DEG sign=UpDown	0,0E+00	7,87
DEG kind=only 1°vsUI	0,0E+00	7,03
CS=7	0,0E+00	5,85

**MFA cluster 2**	p.value	v.test
DM sign=Gain	0,0E+00	16,86
DM kind=notDM	0,0E+00	9,46
DEG sign=Down	0,0E+00	6,92

**MFA cluster 4**	p.value	v.test
DM sign=Gain	0,0E+00	27,14
DM kind=notDM	0,0E+00	12,21
DEG kind=only 1° vs UI	0,0E+00	7,94
DEG sign=Down	0,0E+00	5,75
CS=1	5,0E+07	5,02

**MFA cluster 5**	p.value	v.test
CS=7	0,0E+00	20,18
DM kind=notDM	0,0E+00	11,21
CS=3	0,0E+00	7,42
CS=8	0,0E+00	6,01
DEG kind=only 2° vs PI	0,0E+00	5,58
DM sign=Gain	2,0E+07	5,22

**MFA cluster 9**	p.value	v.test
DM kind=DM	0,0E+00	26,73
DEG kind=not specific	0,0E+00	25,30
DM sign=Gain	0,0E+00	17,65
CS=7	0,0E+00	11,24
DEG sign=Down	0,0E+00	7,74

Figure S7: Multiple Factor Analysis (MFA) input data and output metrics

(A) Groups of variables used for MFA. Groups of variables constitute the MFA input matrix and describe the genes (TOME = Transcriptome, DEG = Differentially expressed gene), the H3K4me2 peaks (Methylome = METH, DMR = Differentially methylated region, EPIG = Chromatin profile, CS = Chromatin state) and their association (DIST = distance, CORR = correlation). Box plots of quantitative variables after normalization and scaling (left). Tables with number of genes/peaks classified according to the differential expression/methylation analysis (right). In total we consider 3874 gene-peaks links for the MFA analysis. **(B)** Quantitative description of factors/dimensions. Cumulative percentage of input dataset variance explained by first five factors (left). Correlation coefficients of groups with the first five factors (right). **(C)** Description of clusters in terms of significant categorical variables. Source Data A-C were deposited into the Gene Expression Omnibus (GEO) repository of the National Center for Biotechnology Information under accession number GSE230142.
