# Supplementary figures and images for: Epithelial cells maintain memory of prior infection with Streptococcus pneumoniae through di-methylation of histone H3
Source: Nat Commun. 2024 Jul 2;15:5545. doi: 10.1038/s41467-024-49347-1 (PMC11219877; doi:10.1038/s41467-024-49347-1)

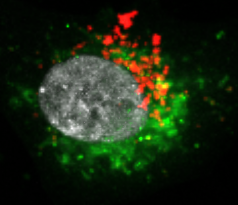

Supplement: Supplementary file 4 — Source Data [file 41467_2024_49347_MOESM4_ESM.zip › Source Data/Figure S2/Images S2.B_LAMP1_Bacteria_Nucleus/FigS2C_2┬░_crop.png]

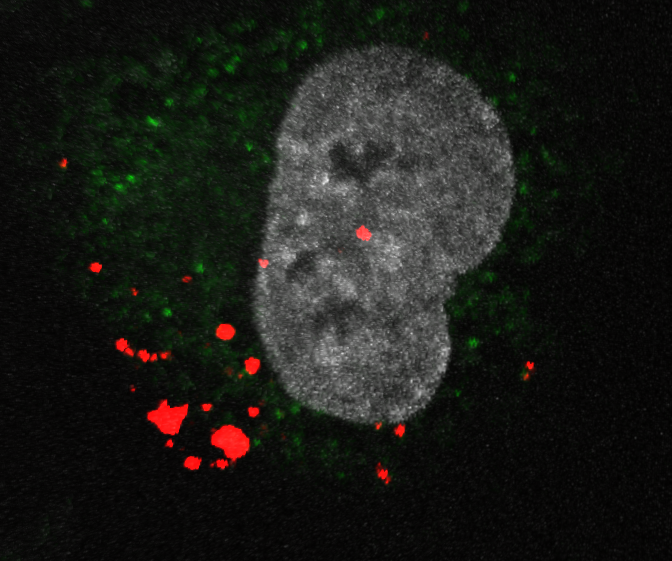

Supplement: Supplementary file 4 — Source Data [file 41467_2024_49347_MOESM4_ESM.zip › Source Data/Figure S2/Images S2.B_LAMP1_Bacteria_Nucleus/FigS2C_1┬░_crop.png]

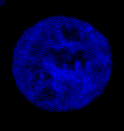

Supplement: Supplementary file 4 — Source Data [file 41467_2024_49347_MOESM4_ESM.zip › Source Data/Figure S4/Fig S4.A_Images H3K4me2 and DAPI in nuclear/1┬░_DAPI_Crop-Composite-1.tif]

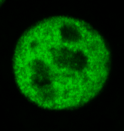

Supplement: Supplementary file 4 — Source Data [file 41467_2024_49347_MOESM4_ESM.zip › Source Data/Figure S4/Fig S4.A_Images H3K4me2 and DAPI in nuclear/1┬░_GFP_Crop_Composite-1.tif]

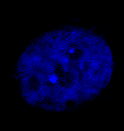

Supplement: Supplementary file 4 — Source Data [file 41467_2024_49347_MOESM4_ESM.zip › Source Data/Figure S4/Fig S4.A_Images H3K4me2 and DAPI in nuclear/PI_DAPI_Crop_Composite.tif]

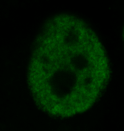

Supplement: Supplementary file 4 — Source Data [file 41467_2024_49347_MOESM4_ESM.zip › Source Data/Figure S4/Fig S4.A_Images H3K4me2 and DAPI in nuclear/UI_GFPCrop_C1-Composite-1.tif]

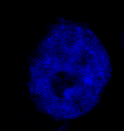

Supplement: Supplementary file 4 — Source Data [file 41467_2024_49347_MOESM4_ESM.zip › Source Data/Figure S4/Fig S4.A_Images H3K4me2 and DAPI in nuclear/UI_DAPI_Crop_C2-Composite-1.tif]

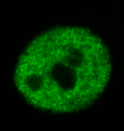

Supplement: Supplementary file 4 — Source Data [file 41467_2024_49347_MOESM4_ESM.zip › Source Data/Figure S4/Fig S4.A_Images H3K4me2 and DAPI in nuclear/PI_GFP_Crop_Composite.tif]

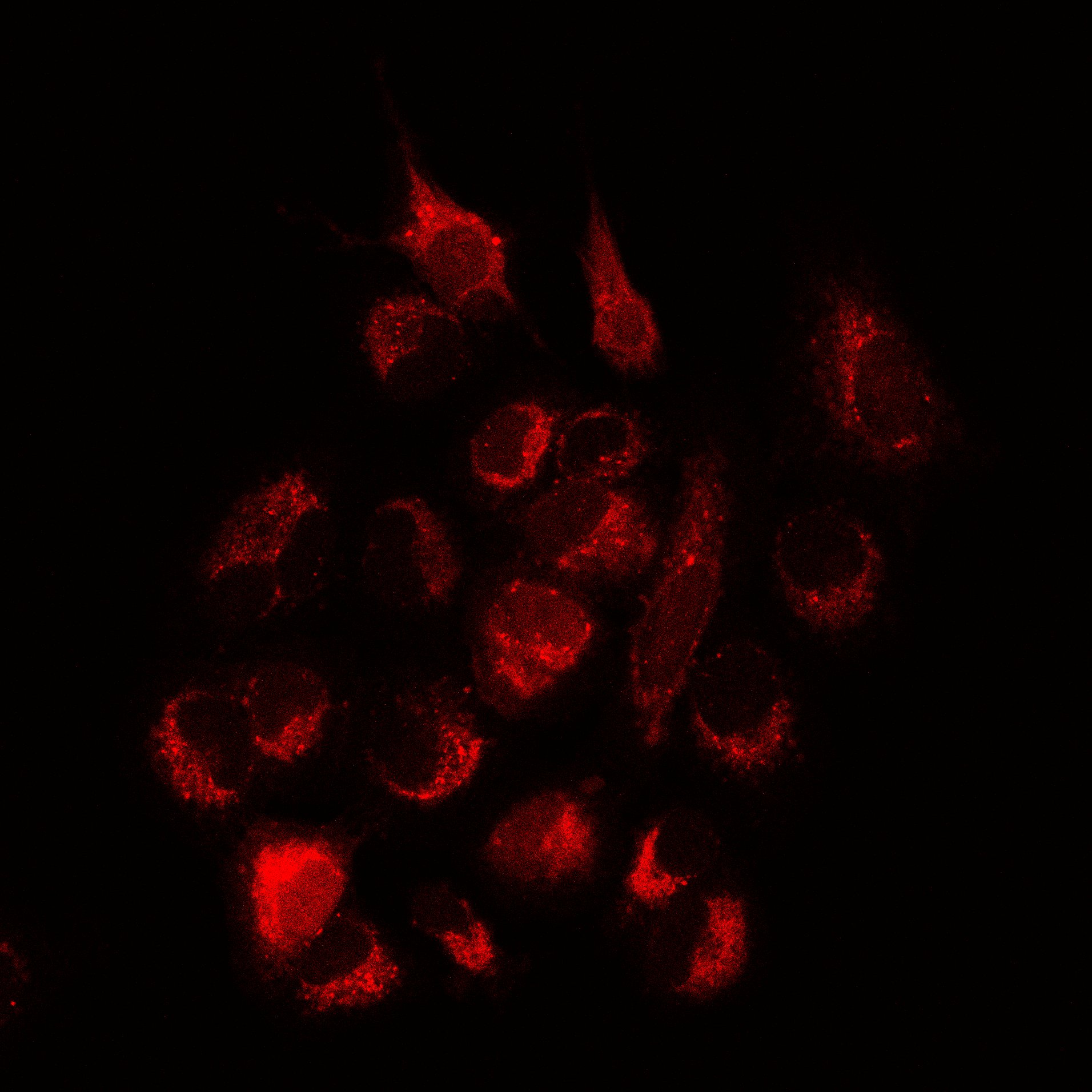

Supplement: Supplementary file 4 — Source Data [file 41467_2024_49347_MOESM4_ESM.zip › Source Data/Figure 2/Fig 2.D_Images_LAMP1_Lysotracker/3h_2┬░_Lysotracker.jpg]

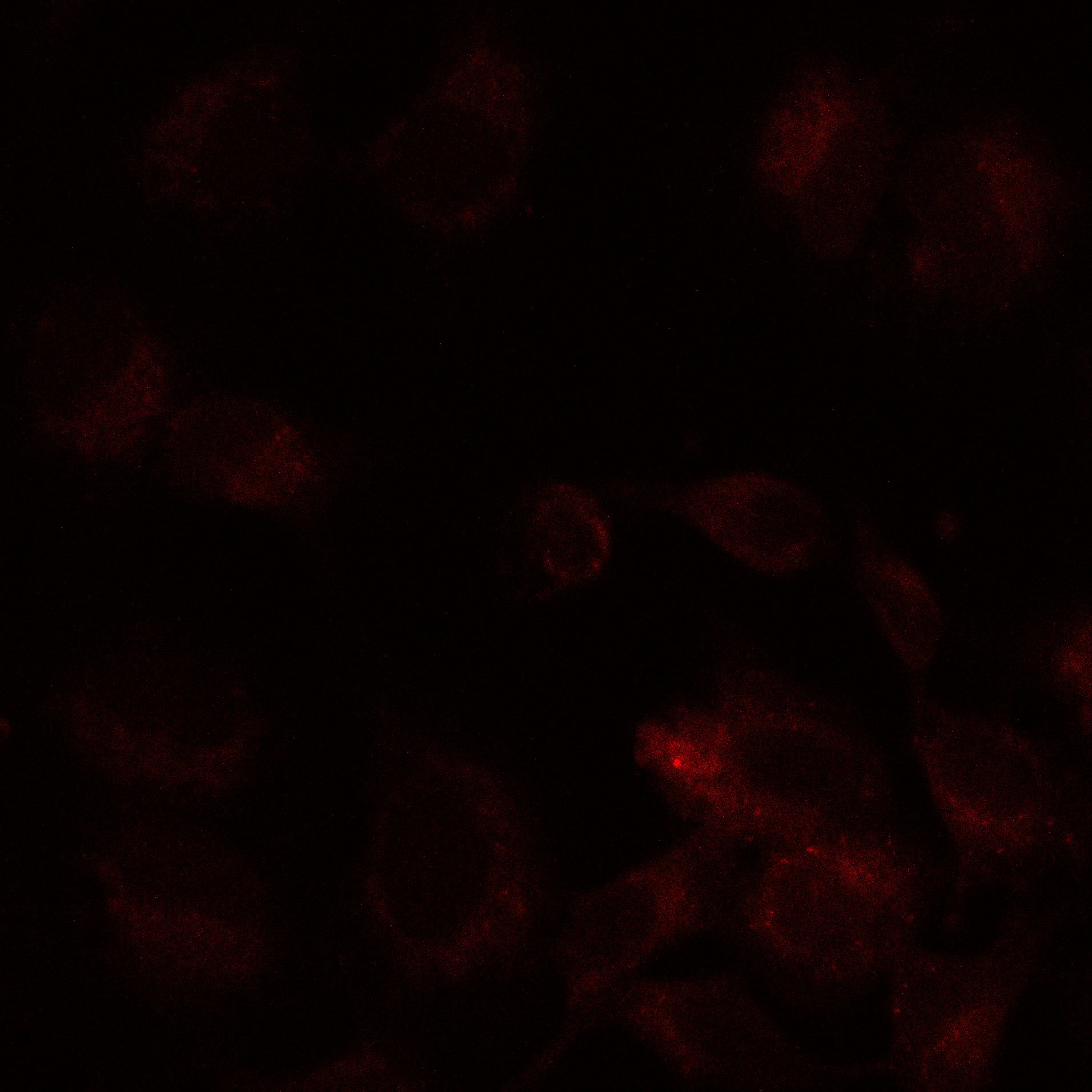

Supplement: Supplementary file 4 — Source Data [file 41467_2024_49347_MOESM4_ESM.zip › Source Data/Figure 2/Fig 2.D_Images_LAMP1_Lysotracker/24h_1┬░_Lysotracker.jpg]

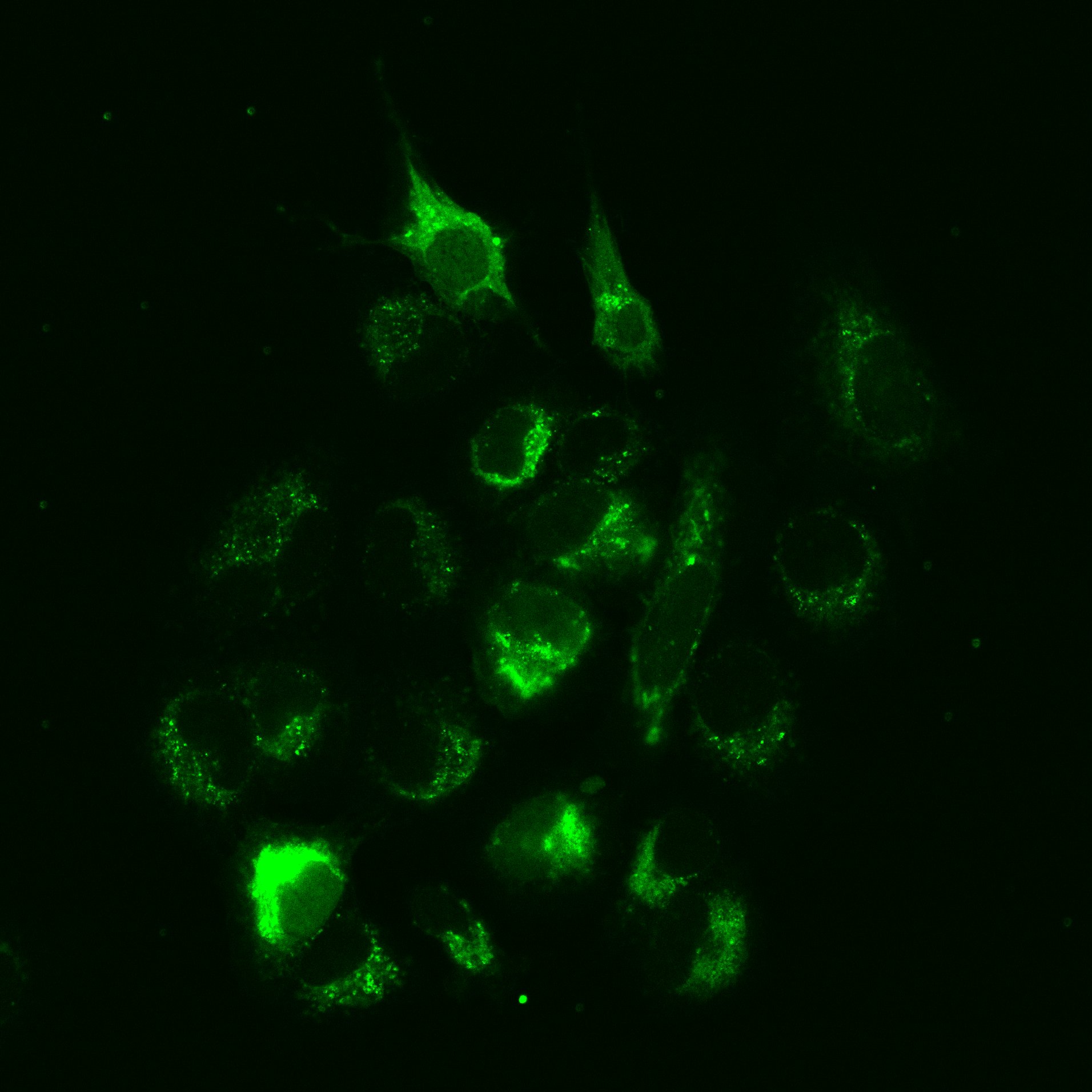

Supplement: Supplementary file 4 — Source Data [file 41467_2024_49347_MOESM4_ESM.zip › Source Data/Figure 2/Fig 2.D_Images_LAMP1_Lysotracker/3h_2┬░_LAMP1.jpg]

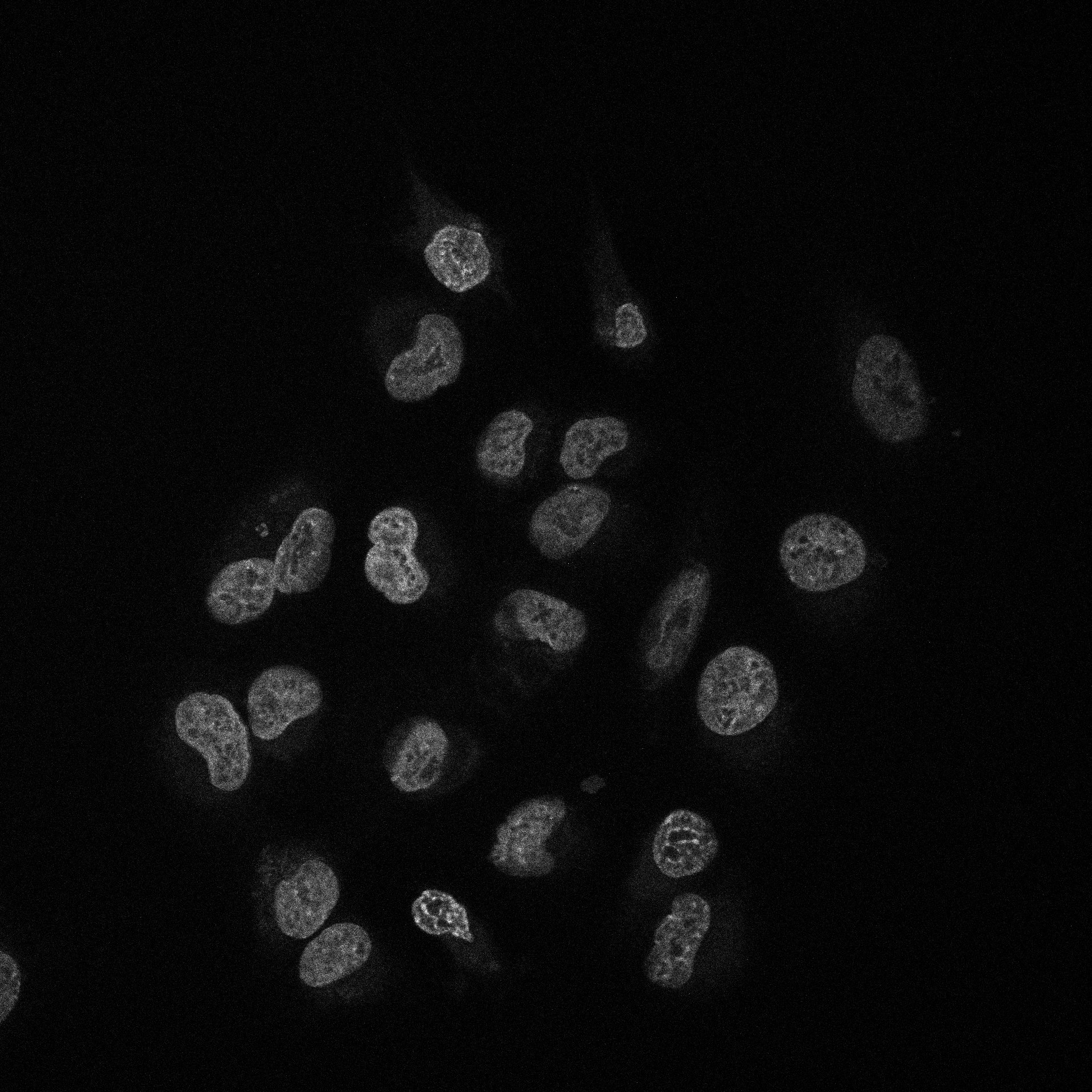

Supplement: Supplementary file 4 — Source Data [file 41467_2024_49347_MOESM4_ESM.zip › Source Data/Figure 2/Fig 2.D_Images_LAMP1_Lysotracker/3h_2┬░_DAPI.jpg]

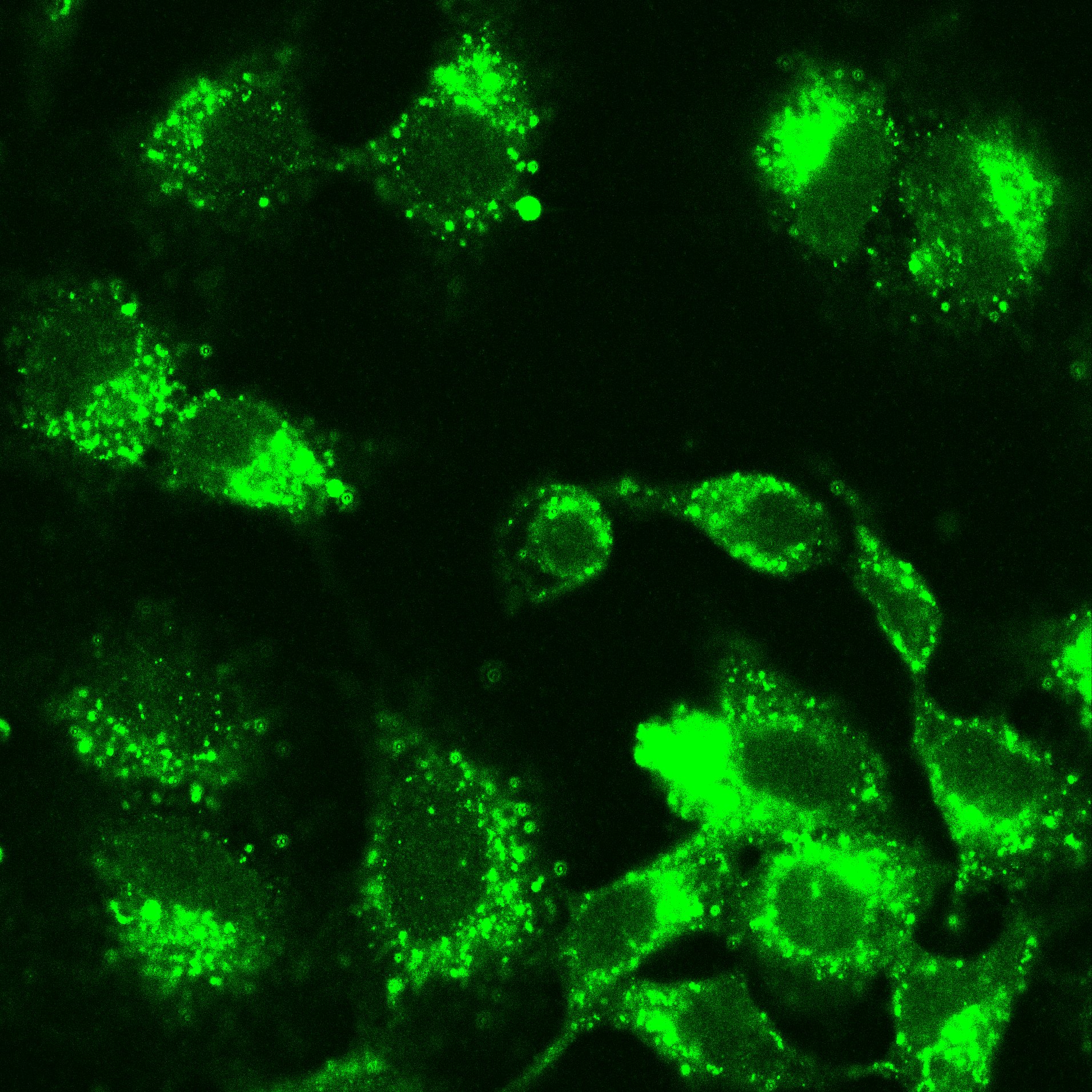

Supplement: Supplementary file 4 — Source Data [file 41467_2024_49347_MOESM4_ESM.zip › Source Data/Figure 2/Fig 2.D_Images_LAMP1_Lysotracker/24h_1┬░_LAMP1.jpg]

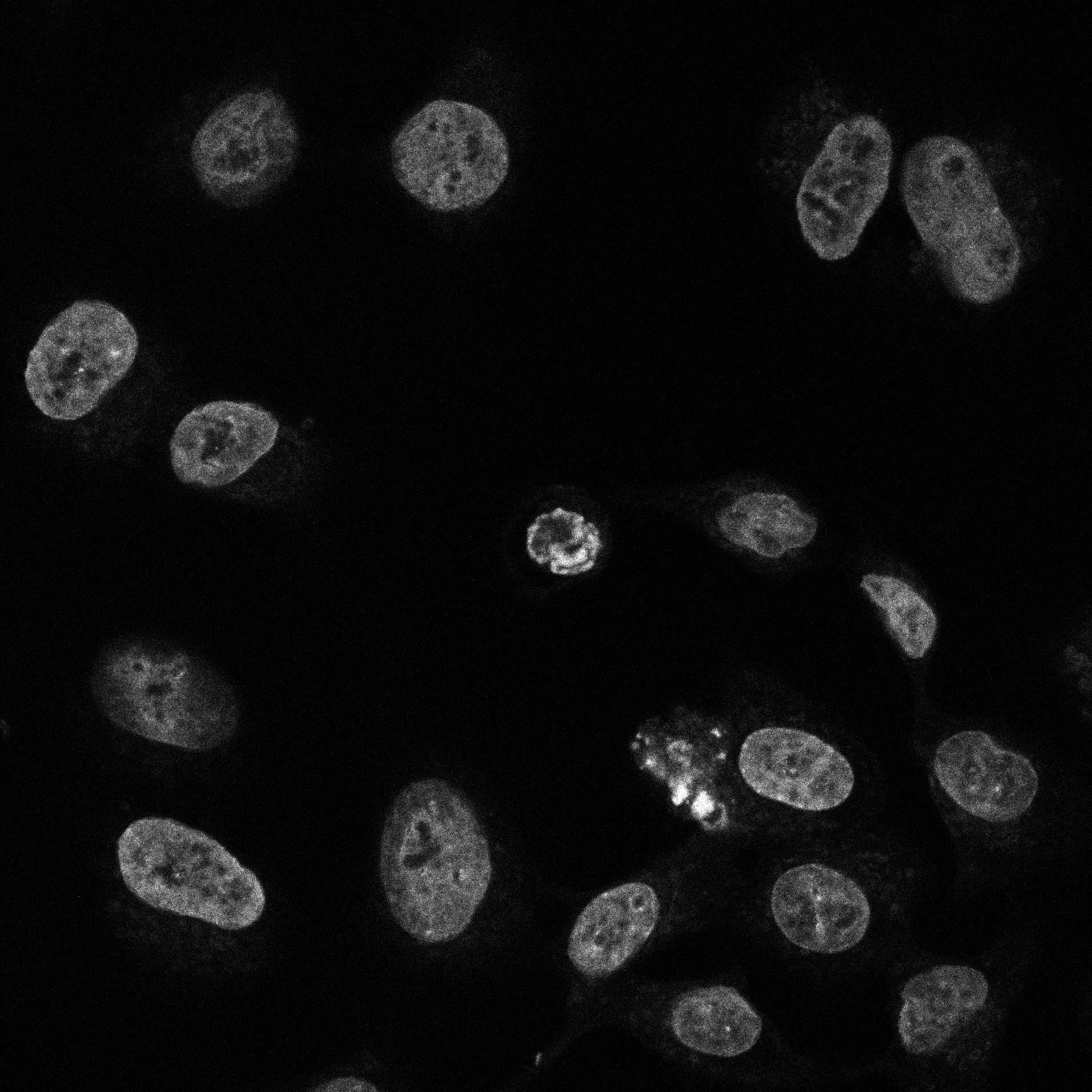

Supplement: Supplementary file 4 — Source Data [file 41467_2024_49347_MOESM4_ESM.zip › Source Data/Figure 2/Fig 2.D_Images_LAMP1_Lysotracker/24h_1┬░_DAPI.jpg]

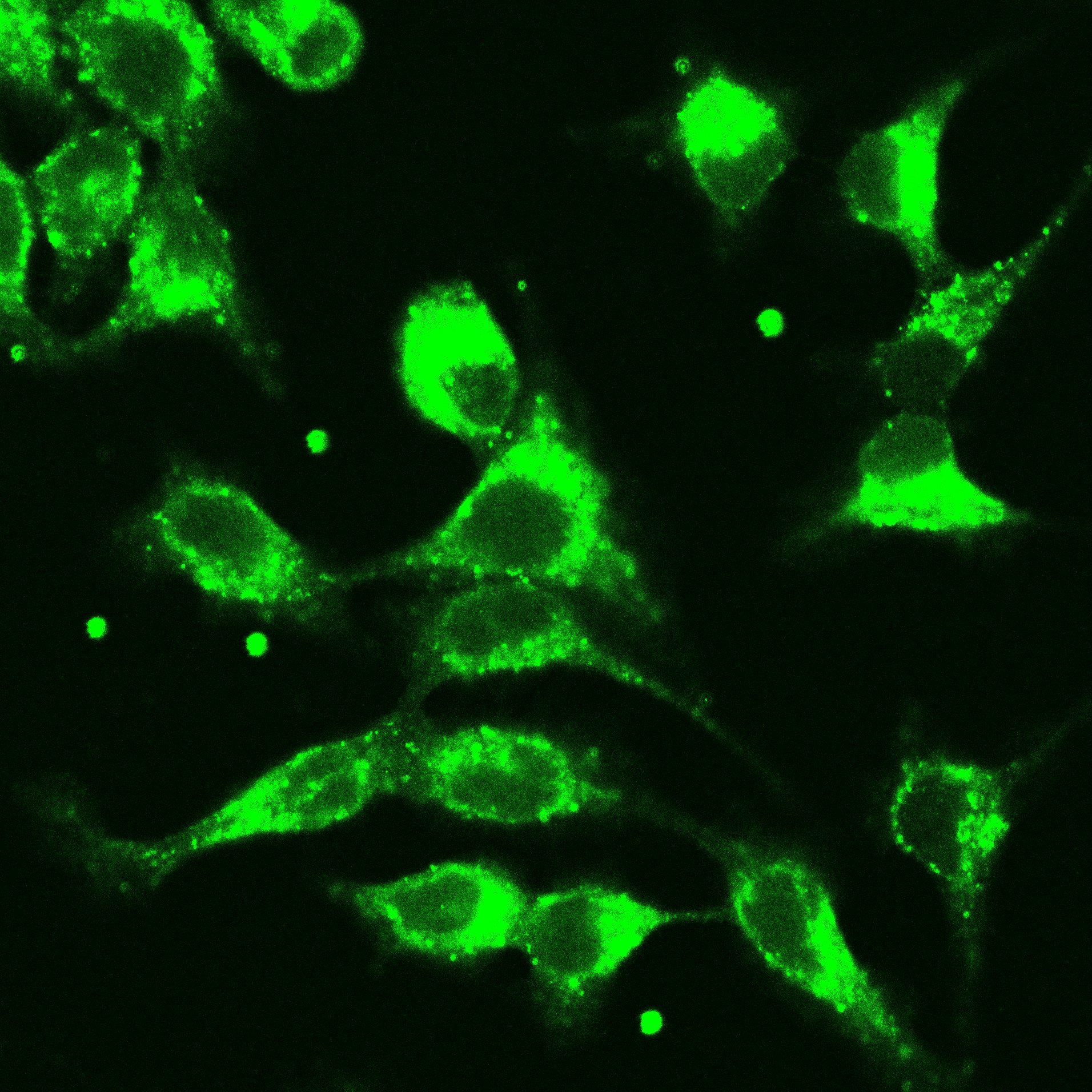

Supplement: Supplementary file 4 — Source Data [file 41467_2024_49347_MOESM4_ESM.zip › Source Data/Figure 2/Fig 2.D_Images_LAMP1_Lysotracker/24h_2┬░_LAMP1.jpg]

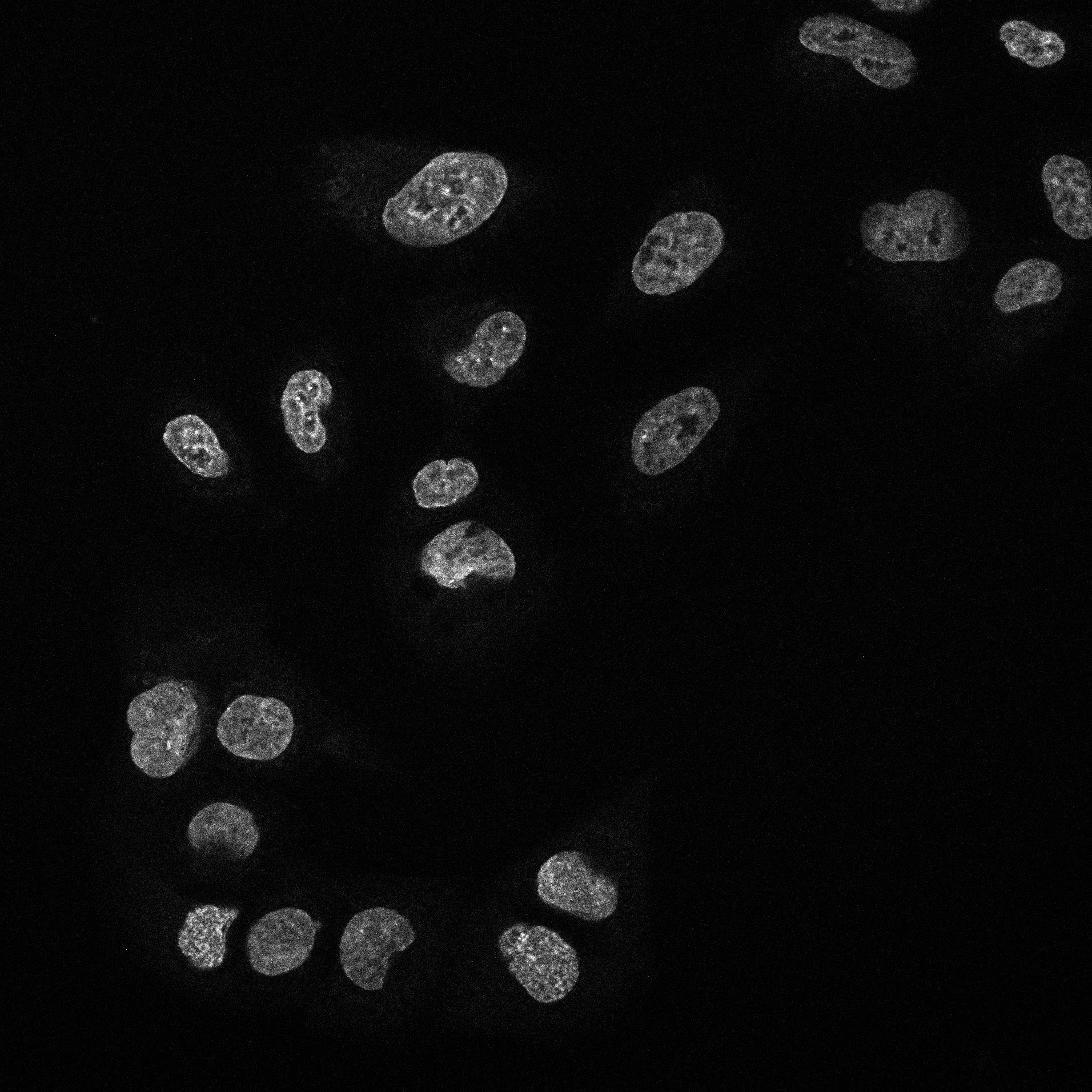

Supplement: Supplementary file 4 — Source Data [file 41467_2024_49347_MOESM4_ESM.zip › Source Data/Figure 2/Fig 2.D_Images_LAMP1_Lysotracker/3h_1┬░_DAPI.jpg]

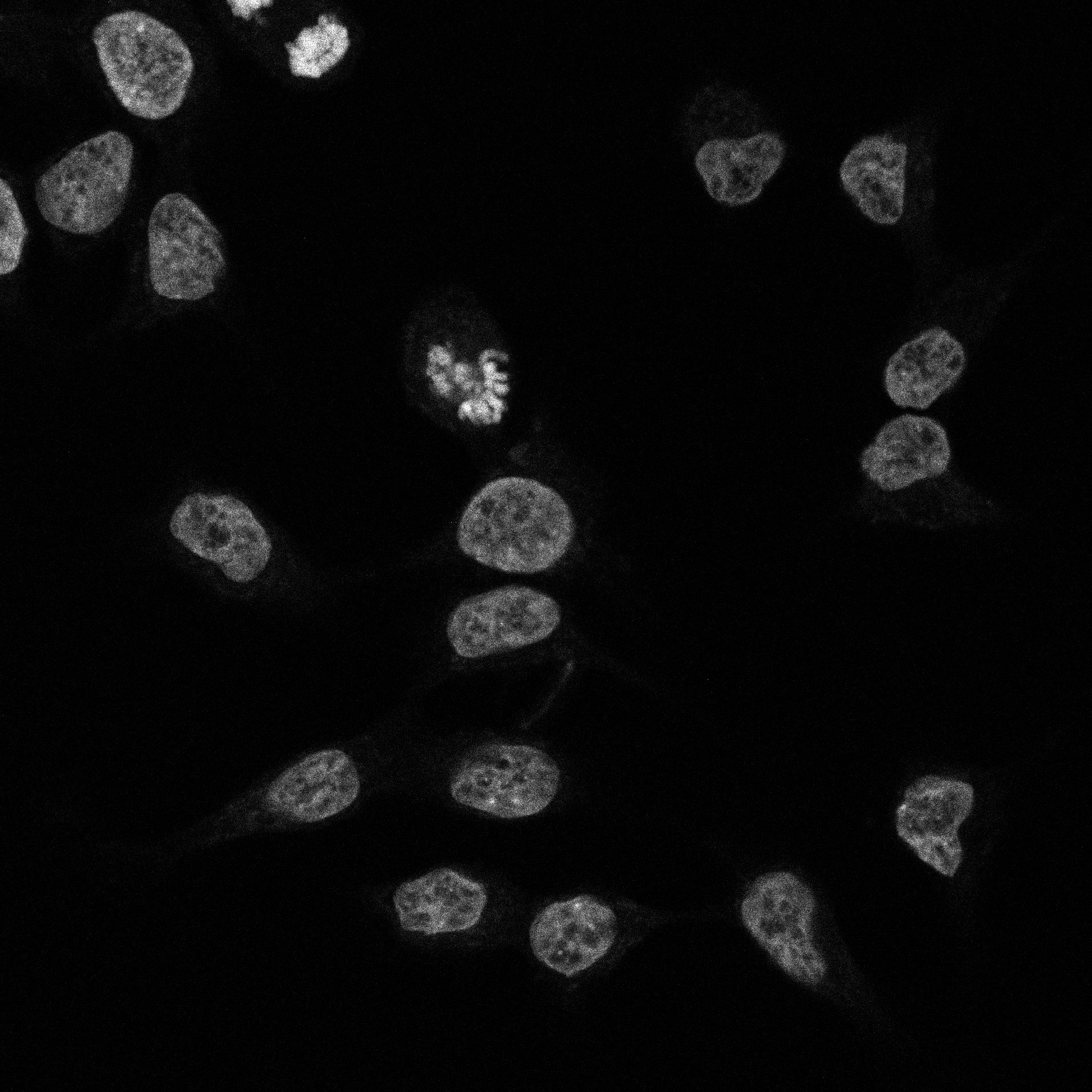

Supplement: Supplementary file 4 — Source Data [file 41467_2024_49347_MOESM4_ESM.zip › Source Data/Figure 2/Fig 2.D_Images_LAMP1_Lysotracker/24h_2┬░_DAPI.jpg]

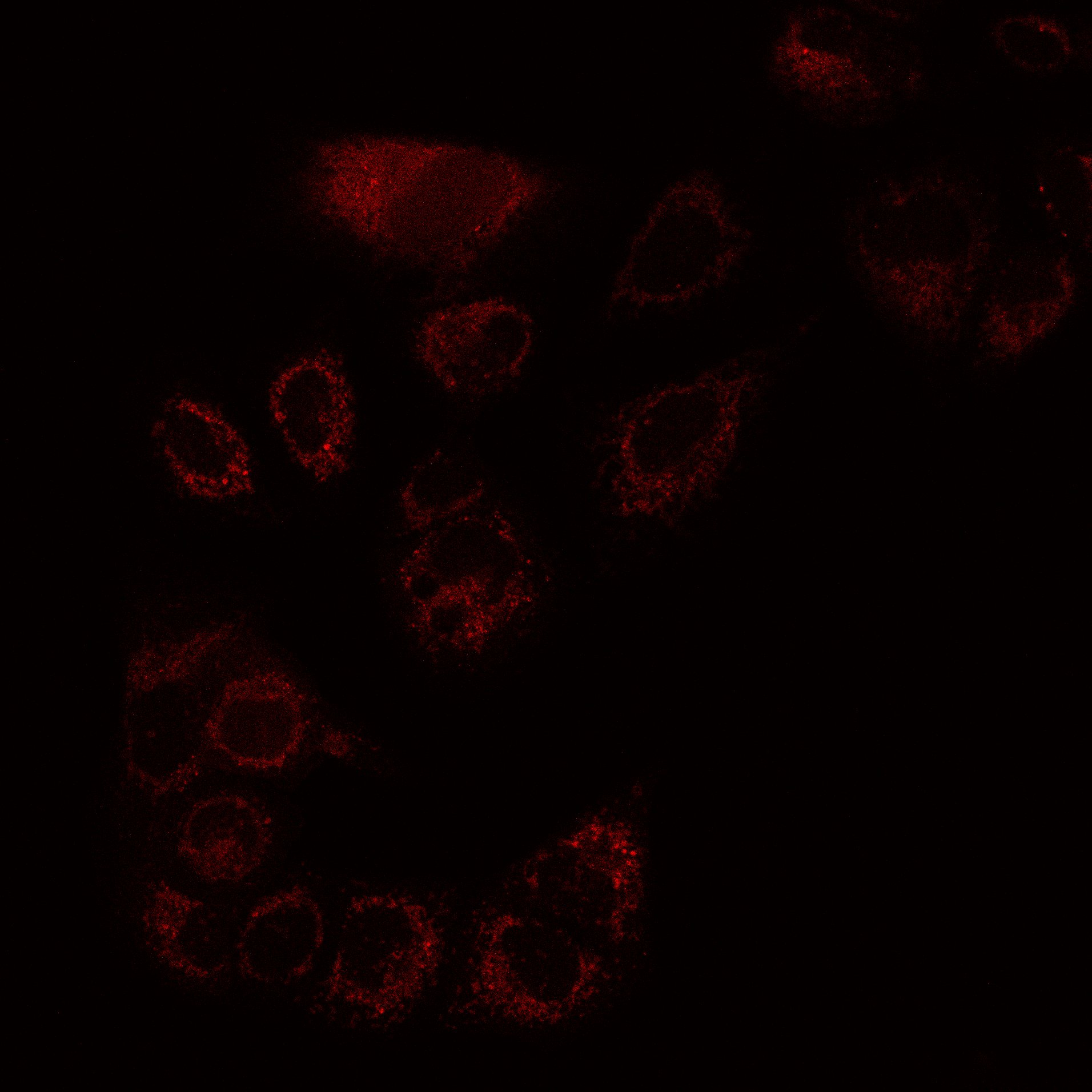

Supplement: Supplementary file 4 — Source Data [file 41467_2024_49347_MOESM4_ESM.zip › Source Data/Figure 2/Fig 2.D_Images_LAMP1_Lysotracker/3h_1┬░_Lysotracker.jpg]

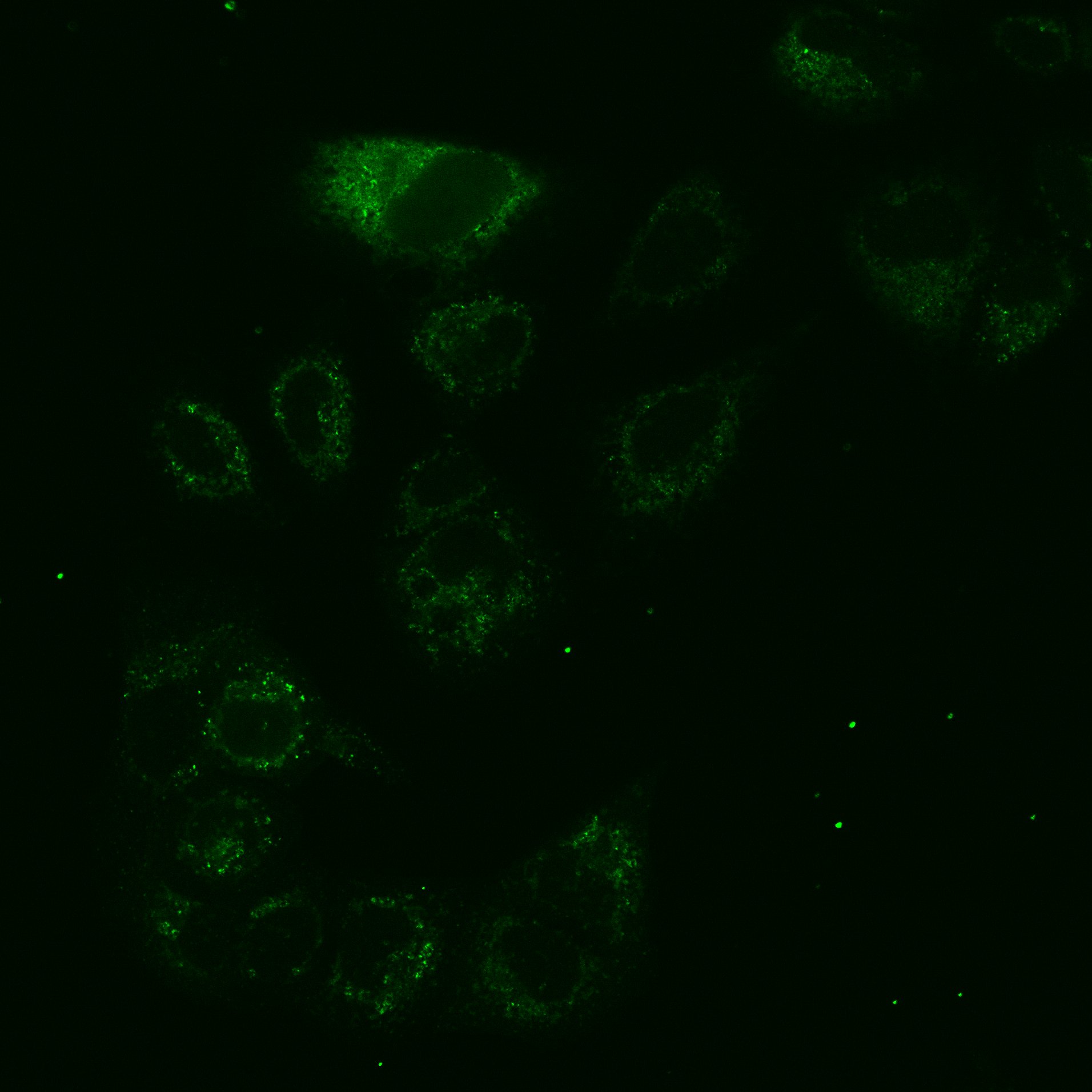

Supplement: Supplementary file 4 — Source Data [file 41467_2024_49347_MOESM4_ESM.zip › Source Data/Figure 2/Fig 2.D_Images_LAMP1_Lysotracker/3h_1┬░_LAMP1.jpg]

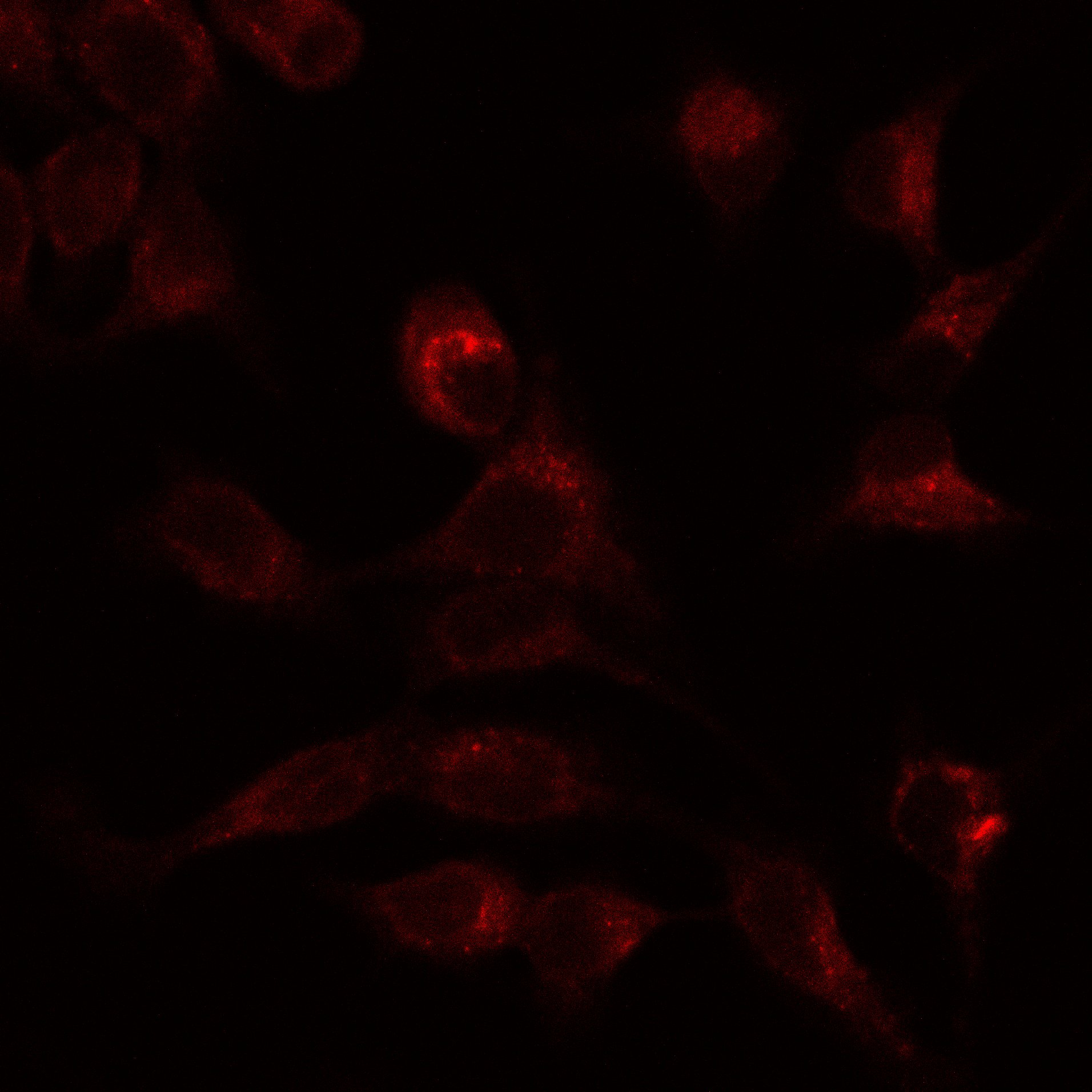

Supplement: Supplementary file 4 — Source Data [file 41467_2024_49347_MOESM4_ESM.zip › Source Data/Figure 2/Fig 2.D_Images_LAMP1_Lysotracker/24h_2┬░_Lysotracker.jpg]

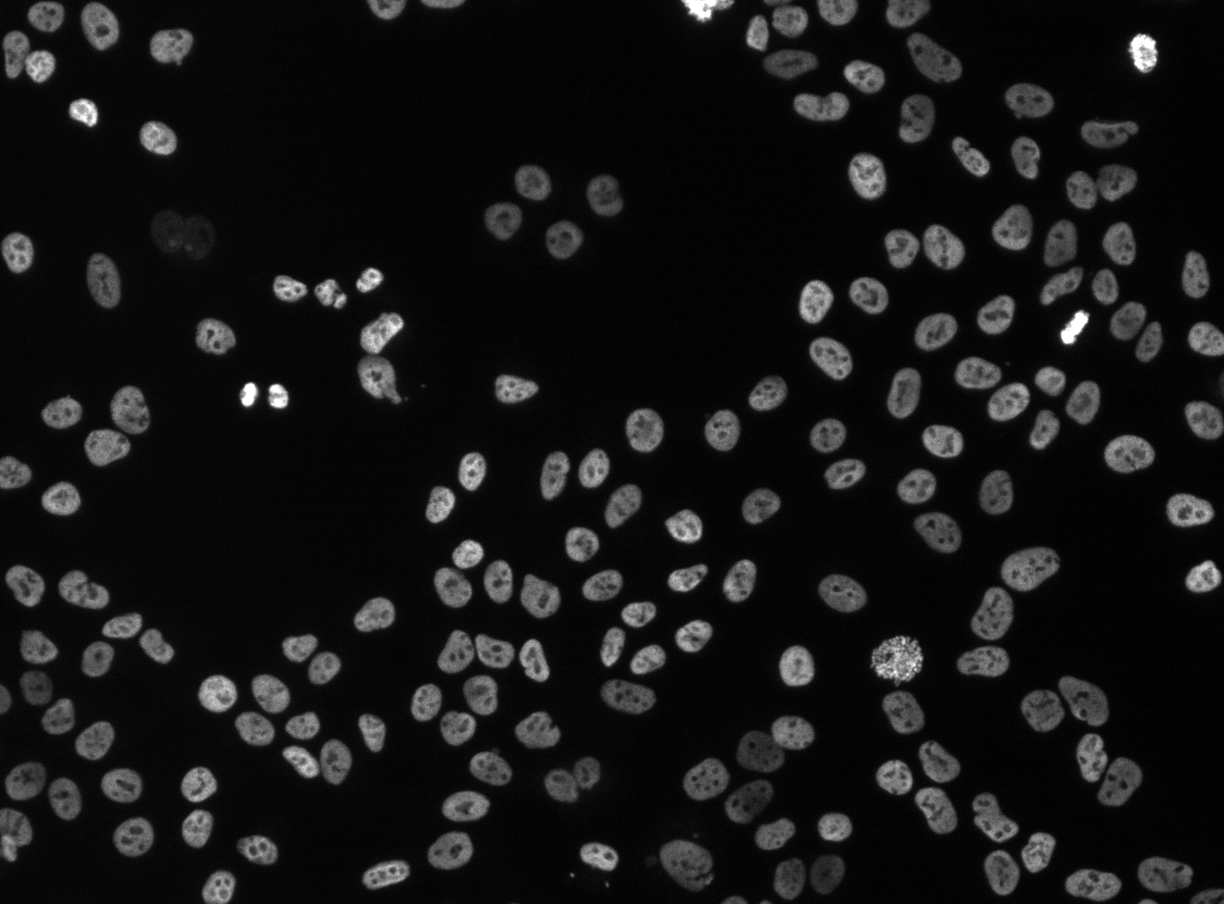

Supplement: Supplementary file 4 — Source Data [file 41467_2024_49347_MOESM4_ESM.zip › Source Data/Figure 4/Fig 4.A_Images Immunofluorescence/UI/A2_01_2_3_GFP_001.tif]

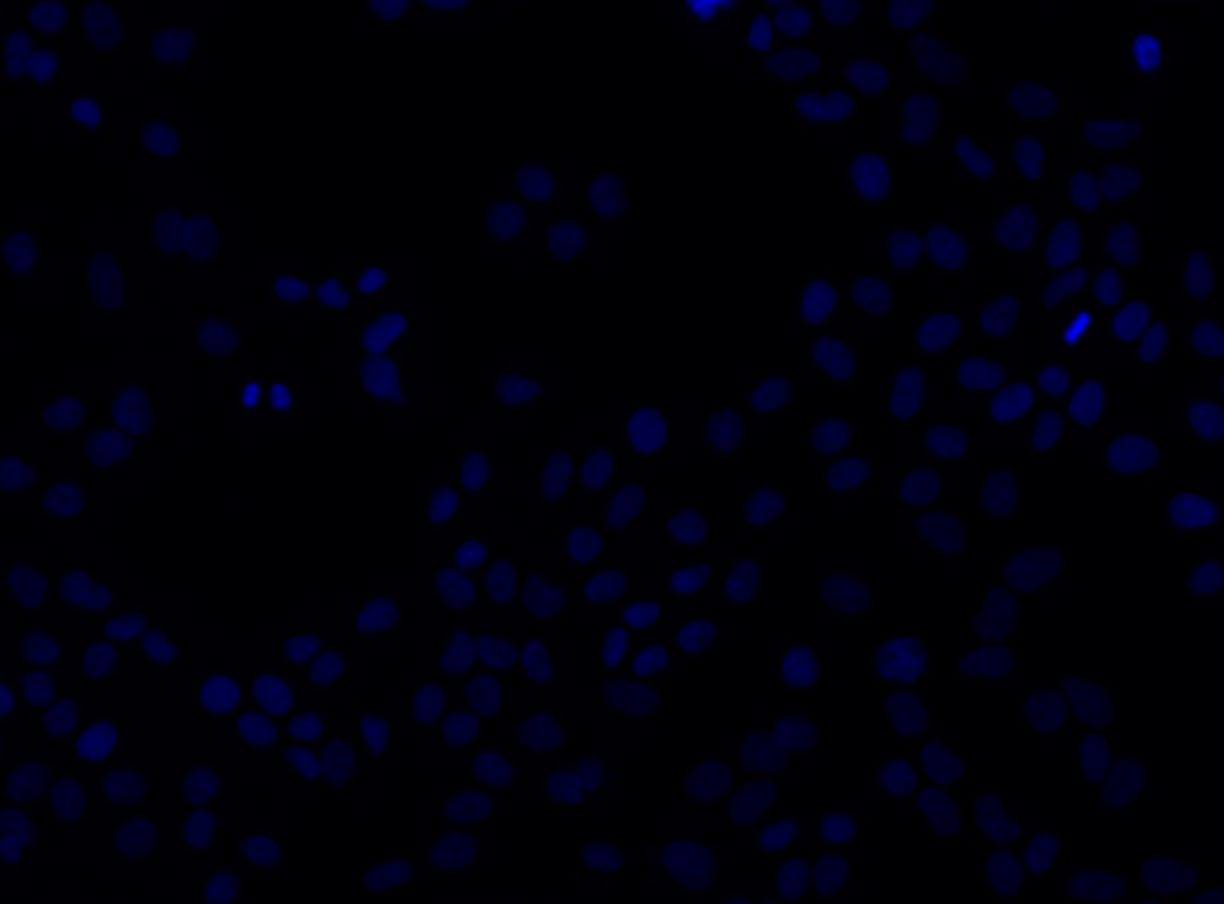

Supplement: Supplementary file 4 — Source Data [file 41467_2024_49347_MOESM4_ESM.zip › Source Data/Figure 4/Fig 4.A_Images Immunofluorescence/UI/DAPI.tif]

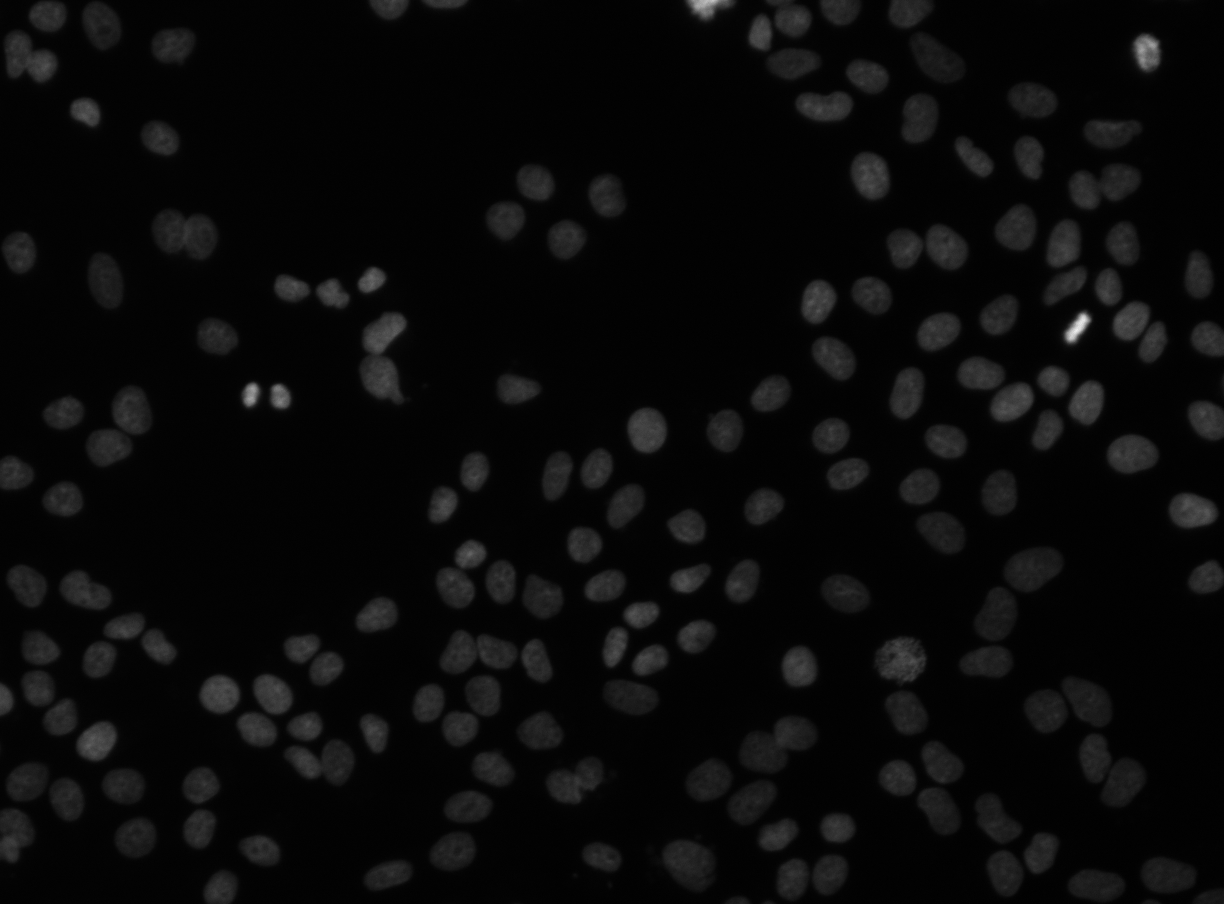

Supplement: Supplementary file 4 — Source Data [file 41467_2024_49347_MOESM4_ESM.zip › Source Data/Figure 4/Fig 4.A_Images Immunofluorescence/UI/A2_01_1_3_DAPI_001.tif]

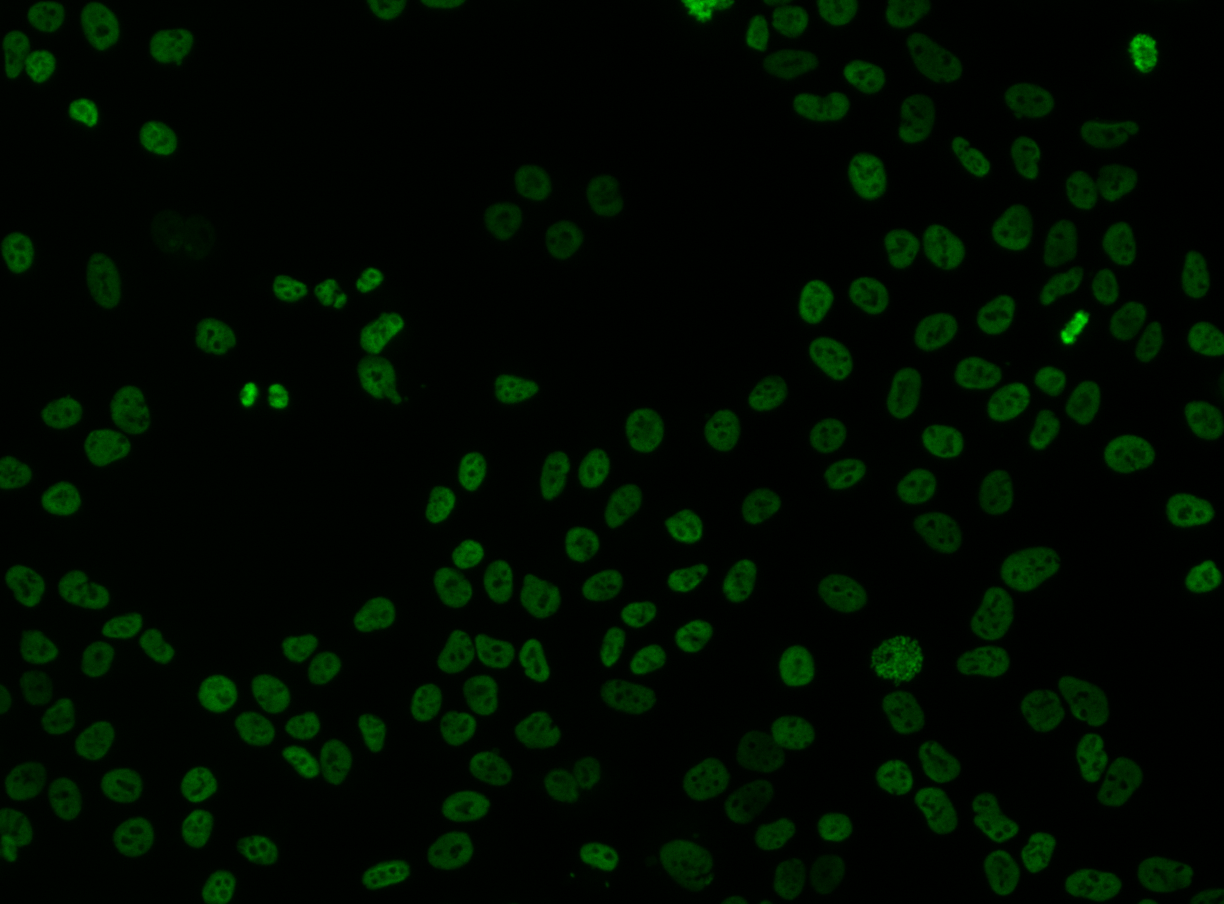

Supplement: Supplementary file 4 — Source Data [file 41467_2024_49347_MOESM4_ESM.zip › Source Data/Figure 4/Fig 4.A_Images Immunofluorescence/UI/H3K4me2_GFP.tif]

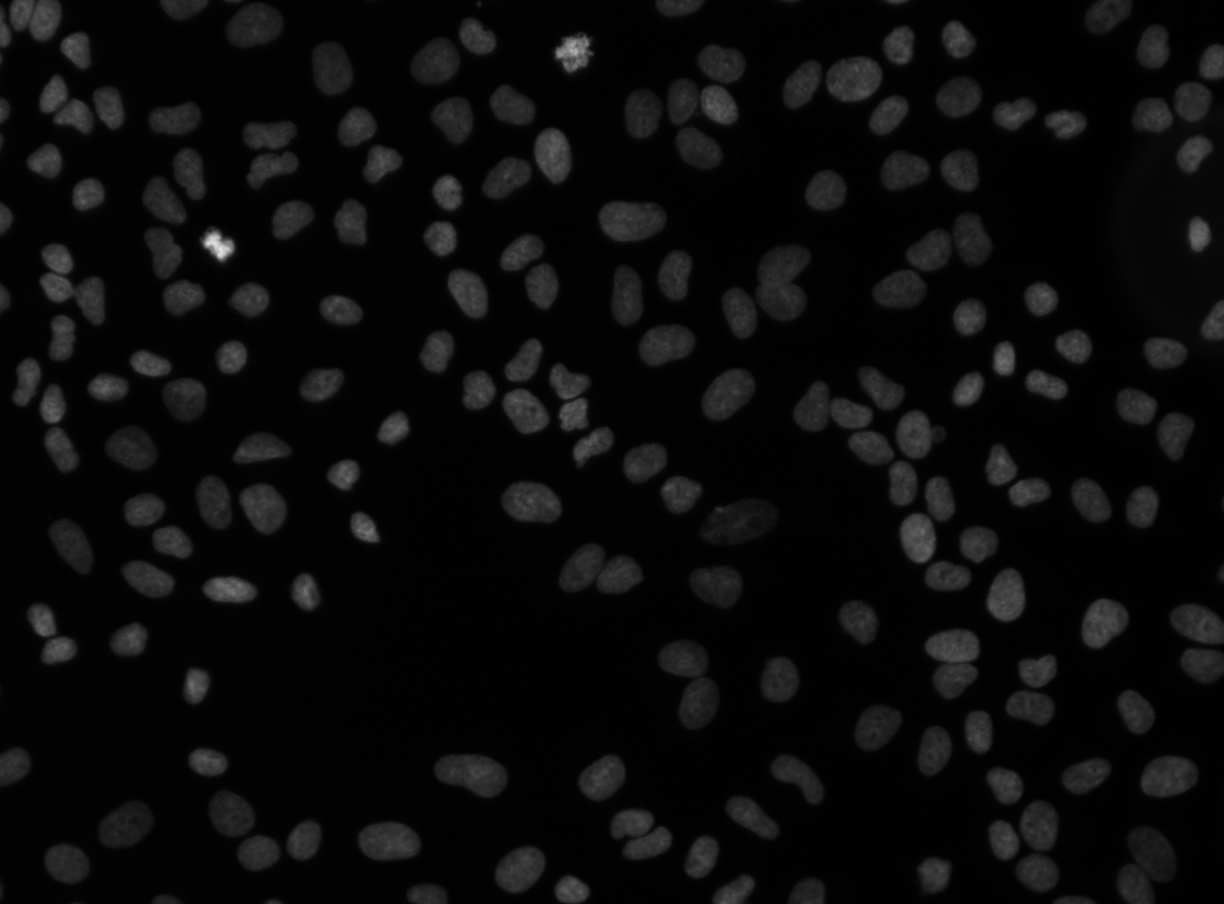

Supplement: Supplementary file 4 — Source Data [file 41467_2024_49347_MOESM4_ESM.zip › Source Data/Figure 4/Fig 4.A_Images Immunofluorescence/Spn inactived_1┬░ /A1_01_1_1_DAPI_001.tif]

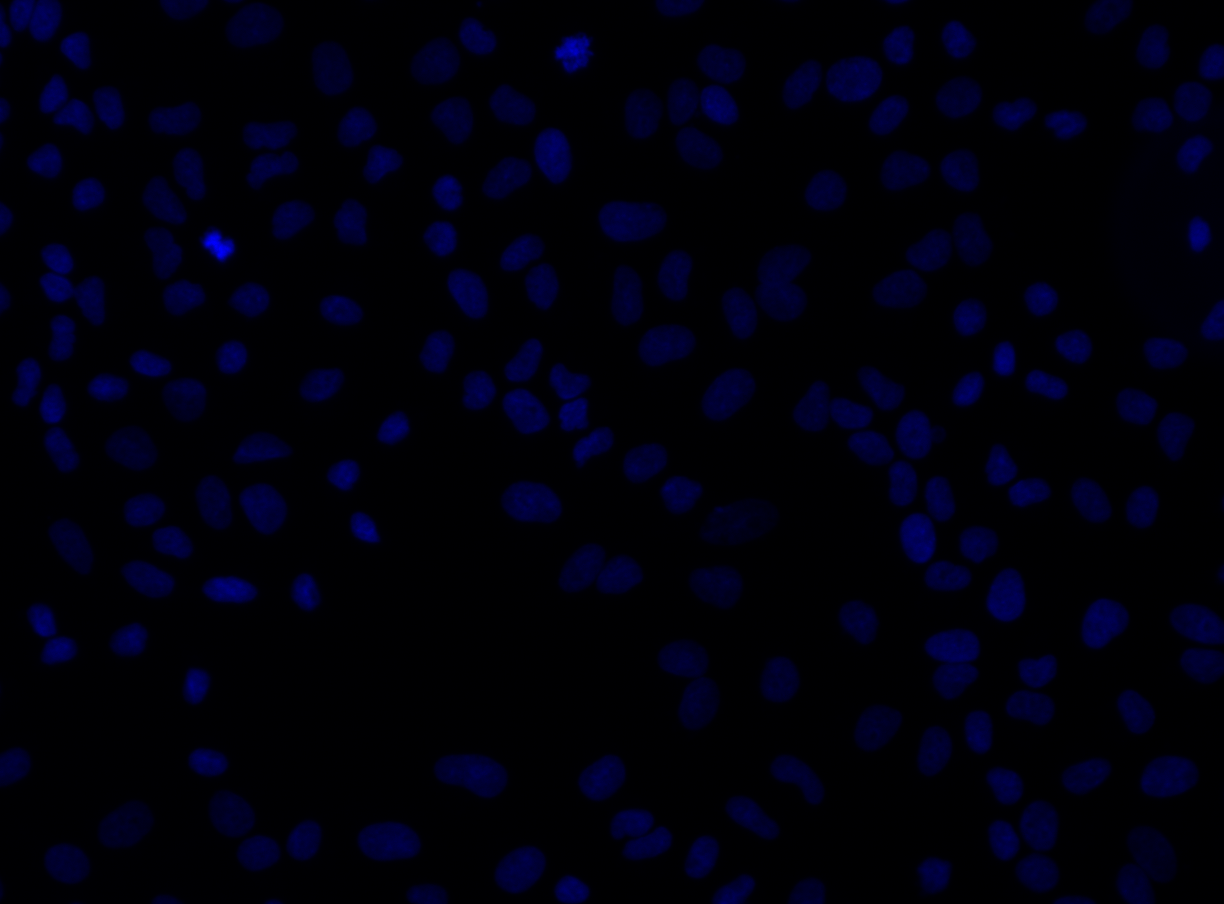

Supplement: Supplementary file 4 — Source Data [file 41467_2024_49347_MOESM4_ESM.zip › Source Data/Figure 4/Fig 4.A_Images Immunofluorescence/Spn inactived_1┬░ /DAPI.tif]

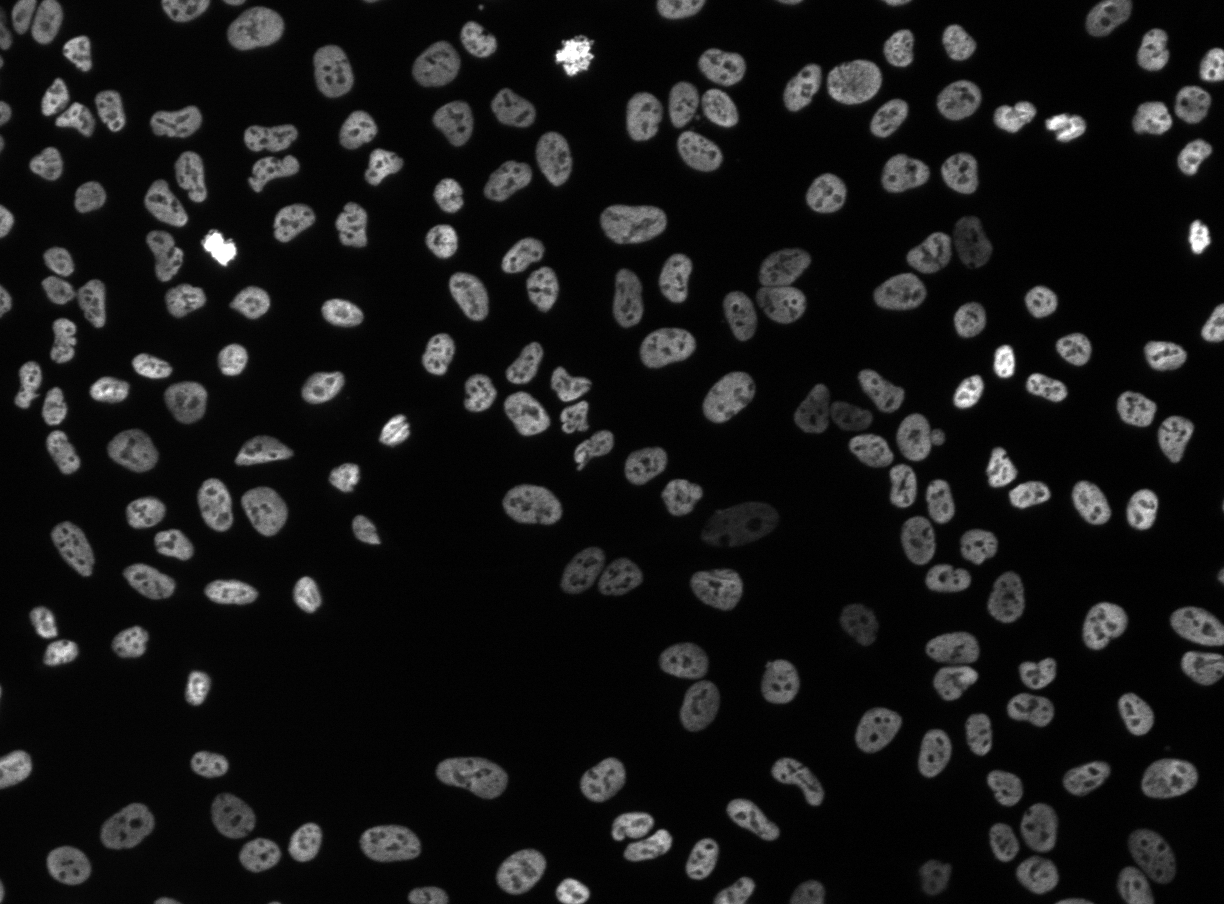

Supplement: Supplementary file 4 — Source Data [file 41467_2024_49347_MOESM4_ESM.zip › Source Data/Figure 4/Fig 4.A_Images Immunofluorescence/Spn inactived_1┬░ /A1_01_2_1_GFP_001.tif]

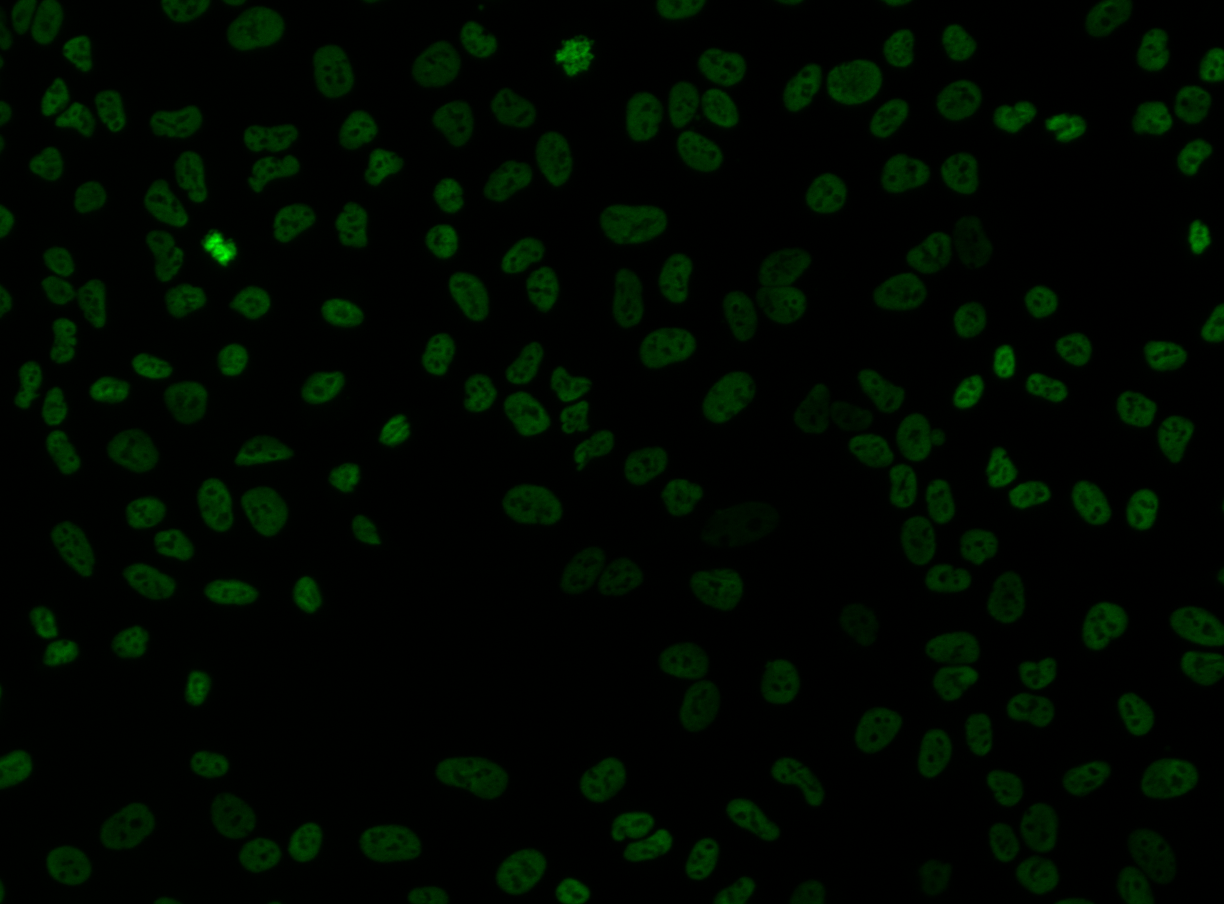

Supplement: Supplementary file 4 — Source Data [file 41467_2024_49347_MOESM4_ESM.zip › Source Data/Figure 4/Fig 4.A_Images Immunofluorescence/Spn inactived_1┬░ /H3K4me2_GFP.tif]

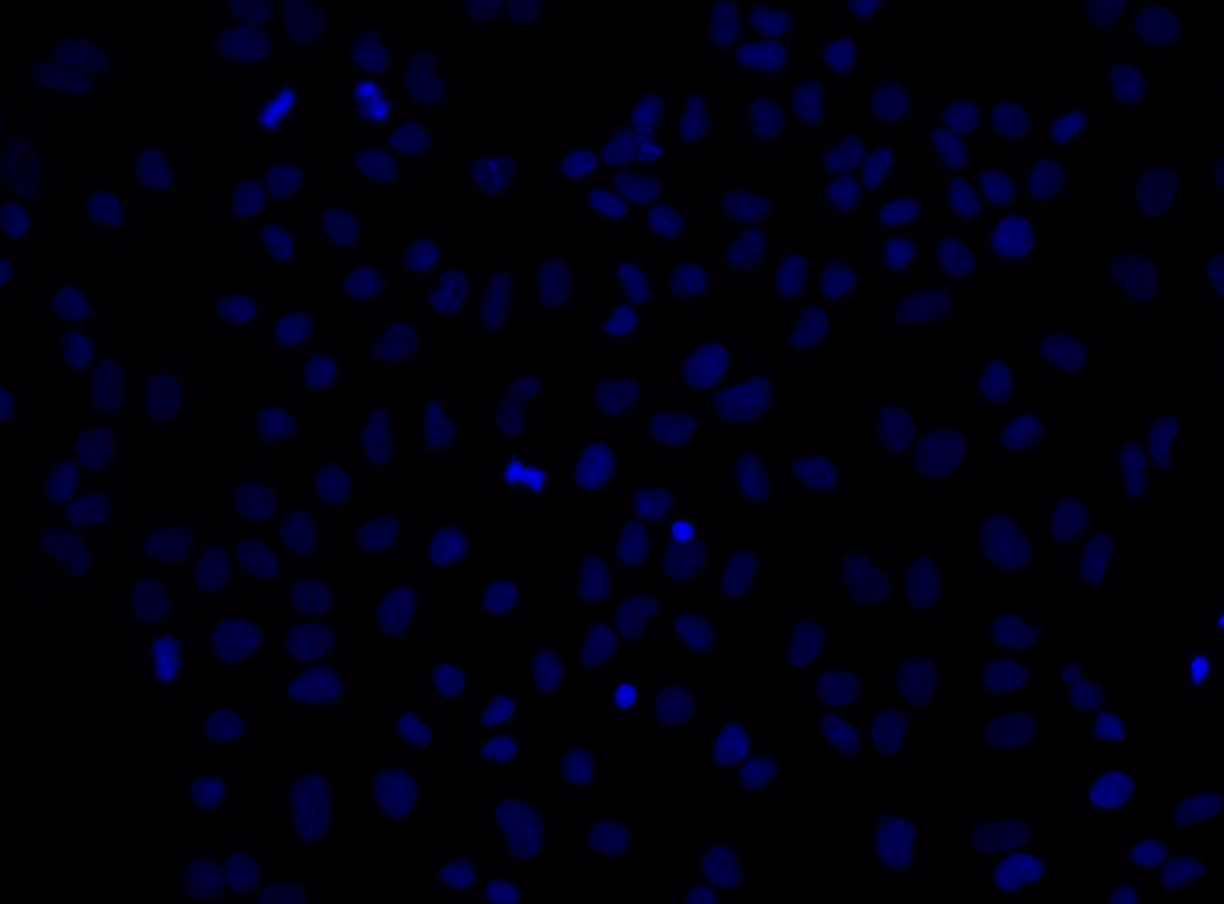

Supplement: Supplementary file 4 — Source Data [file 41467_2024_49347_MOESM4_ESM.zip › Source Data/Figure 4/Fig 4.A_Images Immunofluorescence/Spn live_PI/DAPI.tif]

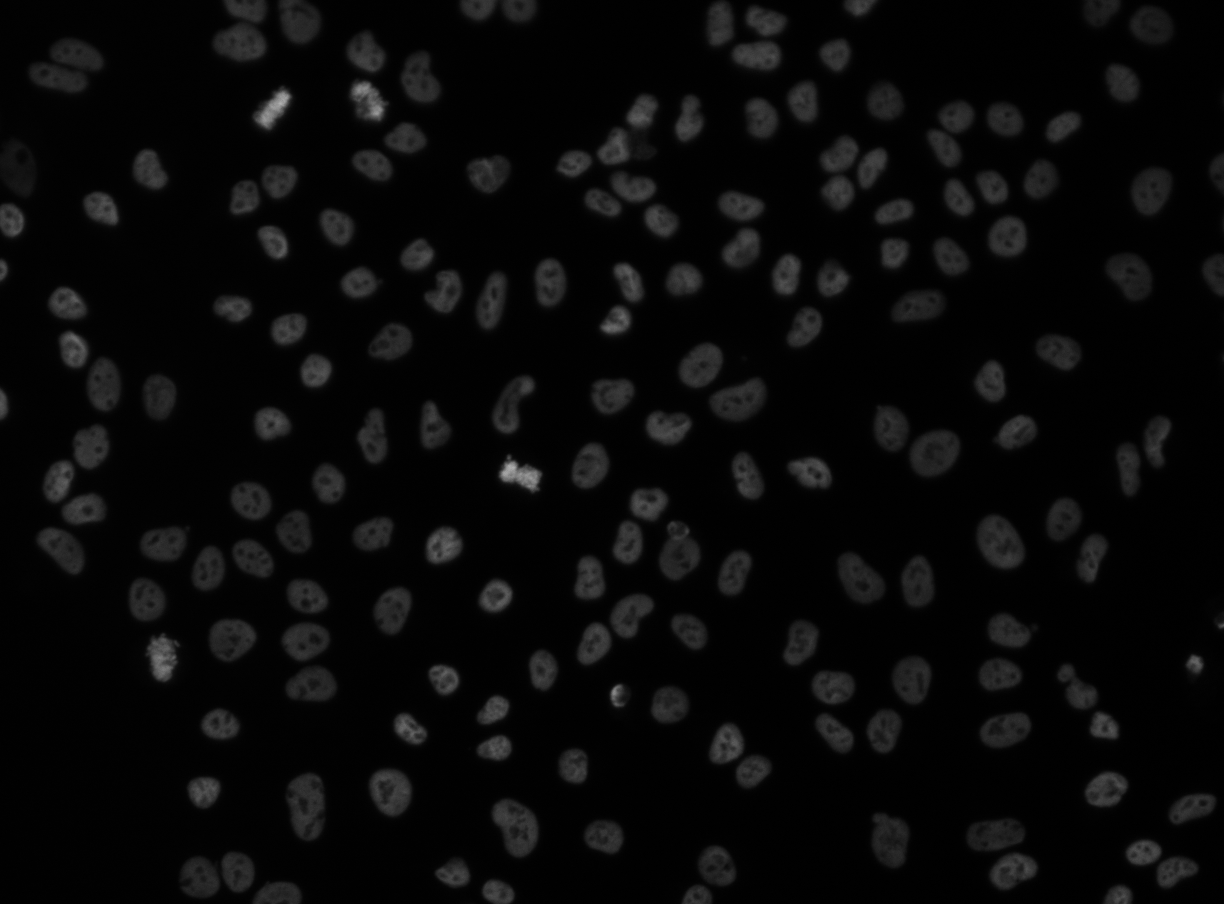

Supplement: Supplementary file 4 — Source Data [file 41467_2024_49347_MOESM4_ESM.zip › Source Data/Figure 4/Fig 4.A_Images Immunofluorescence/Spn live_PI/A1_01_2_4_GFP_001.tif]

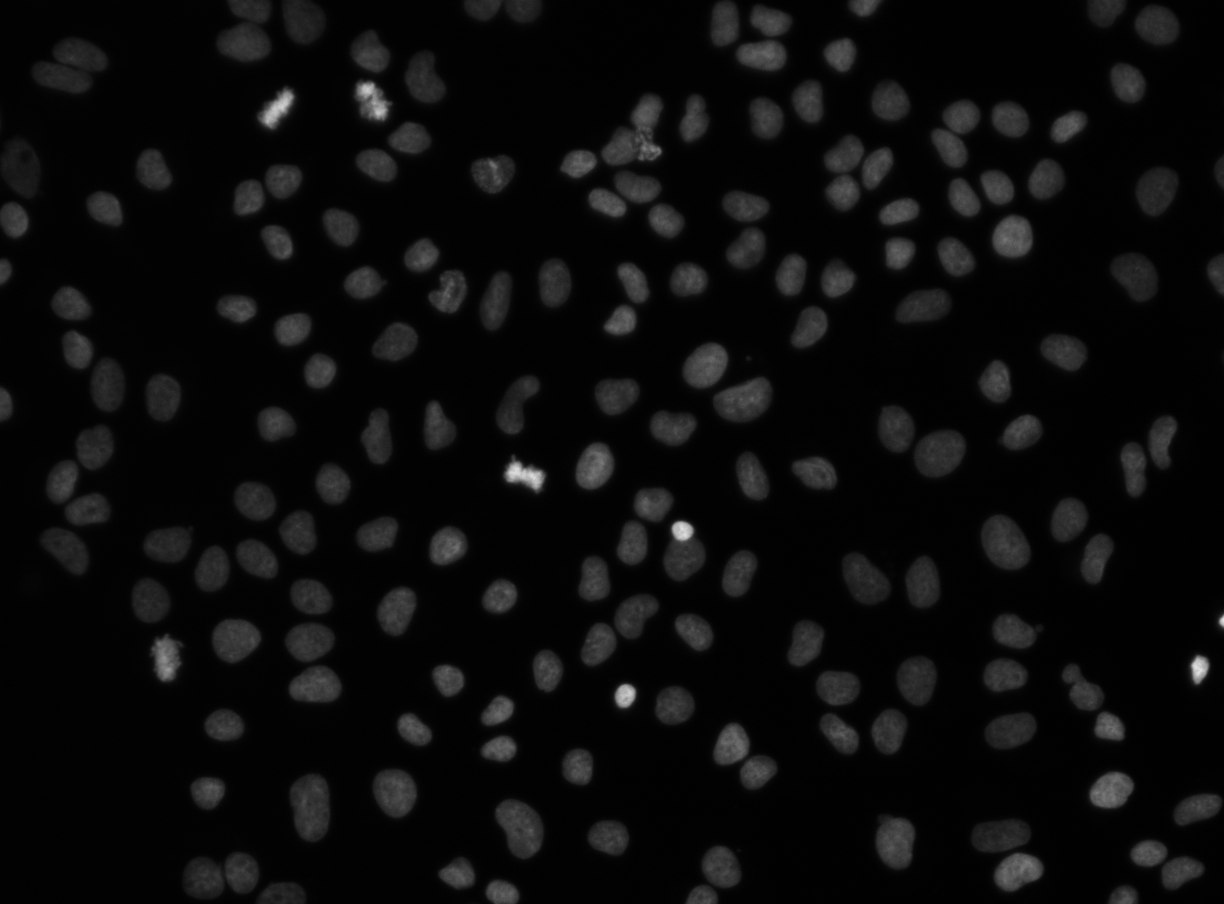

Supplement: Supplementary file 4 — Source Data [file 41467_2024_49347_MOESM4_ESM.zip › Source Data/Figure 4/Fig 4.A_Images Immunofluorescence/Spn live_PI/A1_01_1_4_DAPI_001.tif]

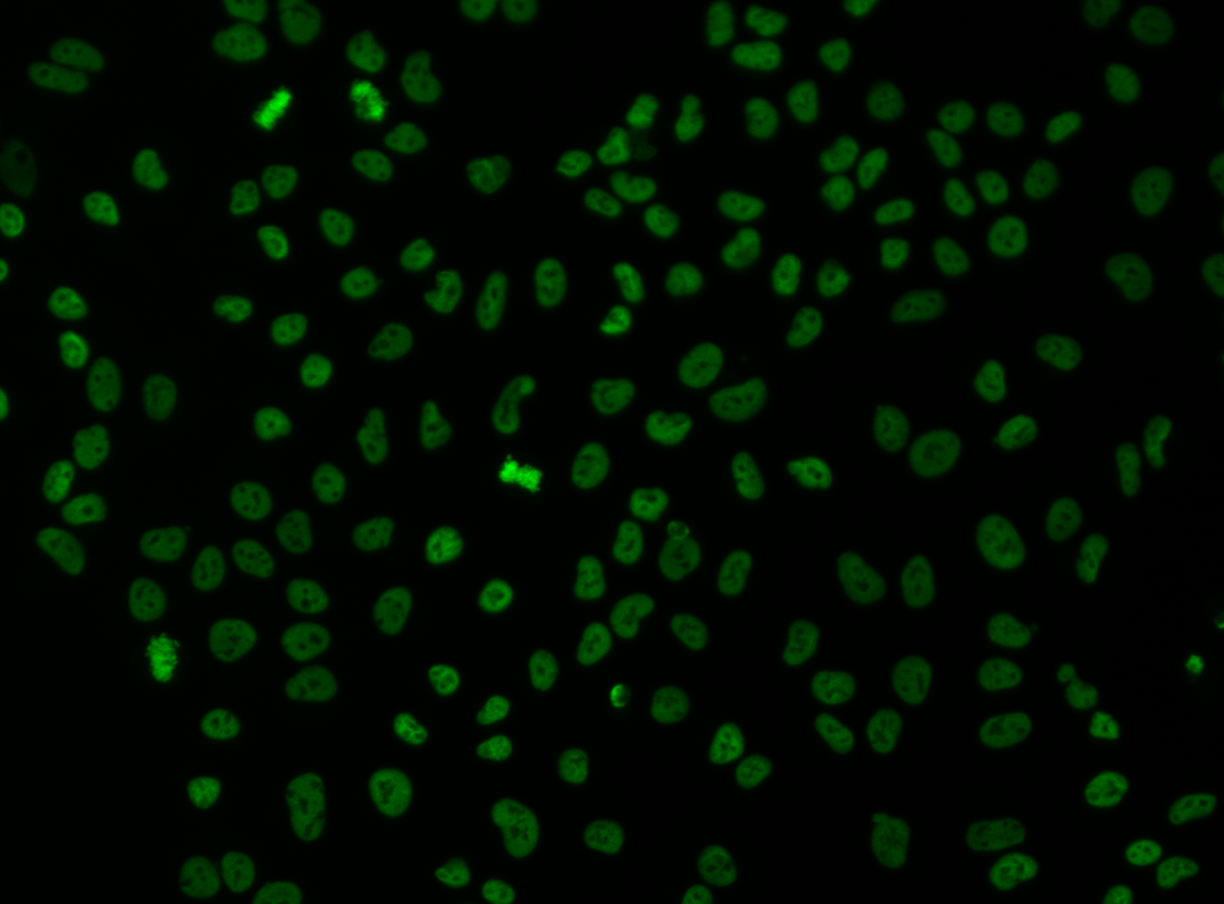

Supplement: Supplementary file 4 — Source Data [file 41467_2024_49347_MOESM4_ESM.zip › Source Data/Figure 4/Fig 4.A_Images Immunofluorescence/Spn live_PI/H3K4me2_GFP.tif]

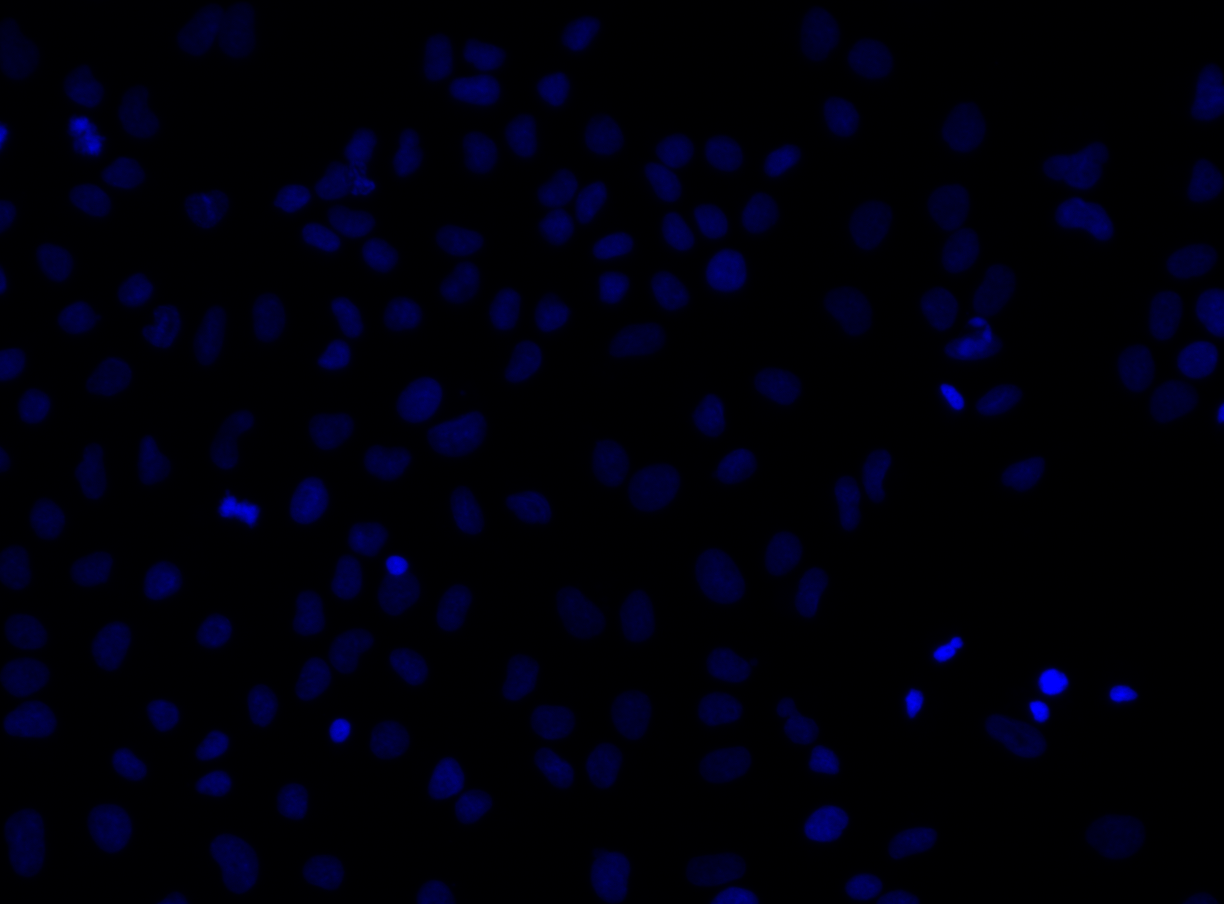

Supplement: Supplementary file 4 — Source Data [file 41467_2024_49347_MOESM4_ESM.zip › Source Data/Figure 4/Fig 4.A_Images Immunofluorescence/Spn live_1┬░/DAPI.tif]

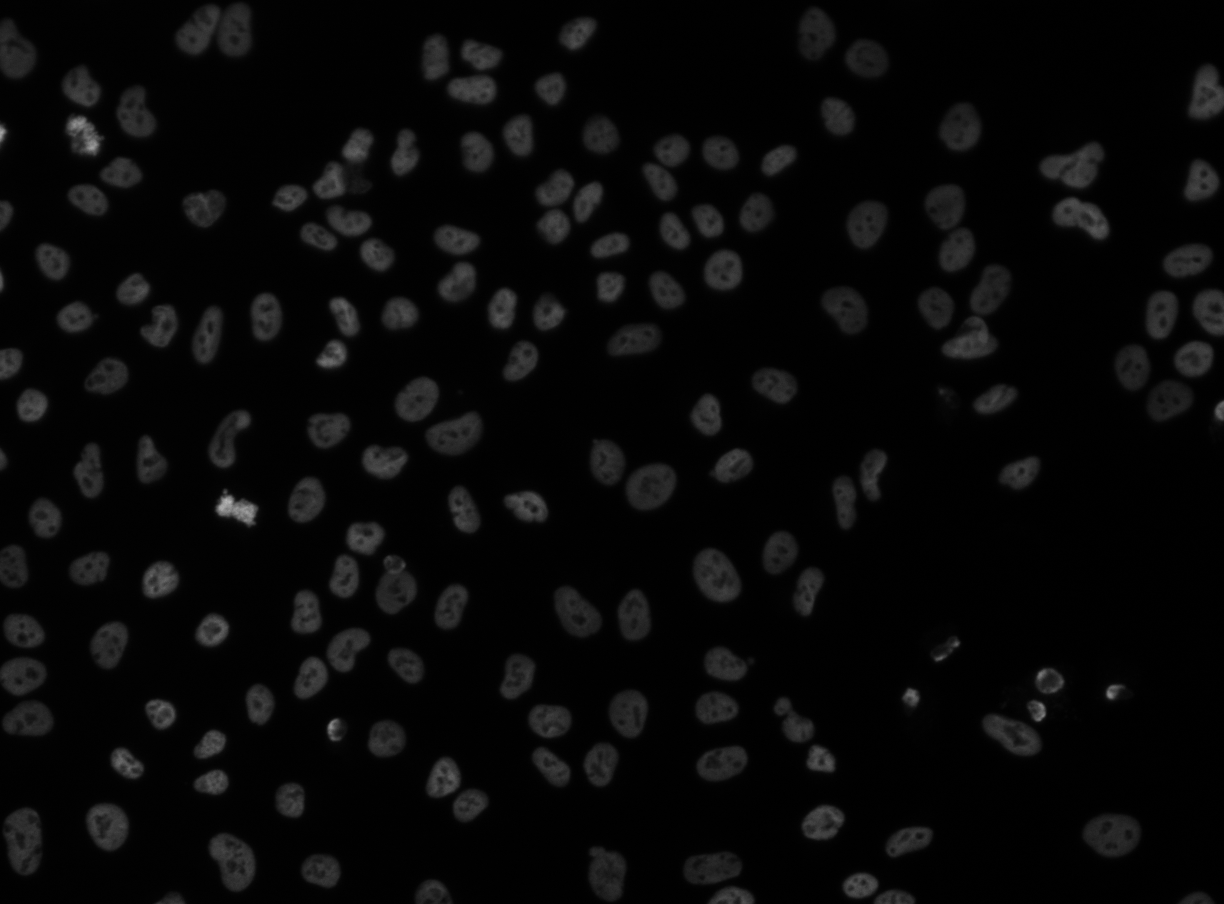

Supplement: Supplementary file 4 — Source Data [file 41467_2024_49347_MOESM4_ESM.zip › Source Data/Figure 4/Fig 4.A_Images Immunofluorescence/Spn live_1┬░/A1_01_2_4_GFP_001.tif]

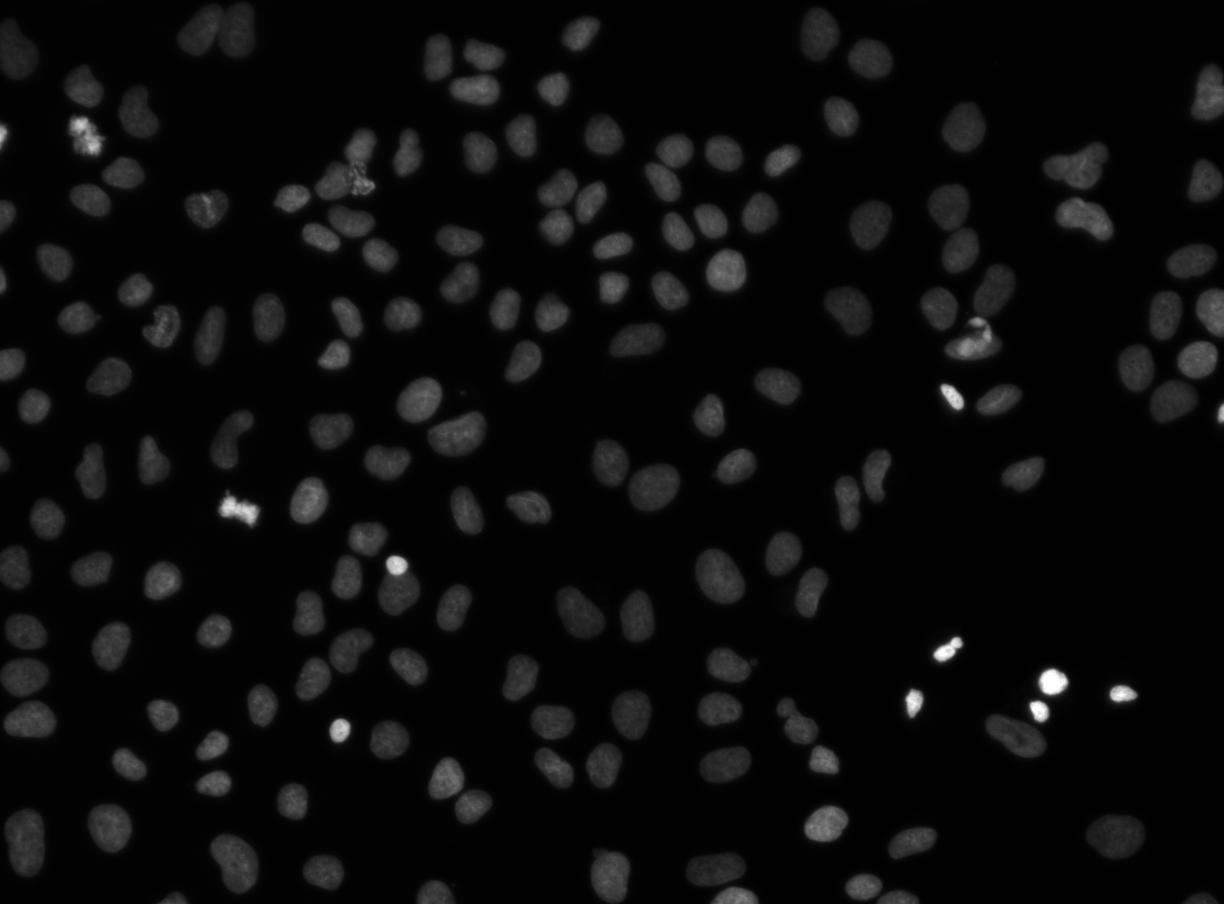

Supplement: Supplementary file 4 — Source Data [file 41467_2024_49347_MOESM4_ESM.zip › Source Data/Figure 4/Fig 4.A_Images Immunofluorescence/Spn live_1┬░/A1_01_1_4_DAPI_001.tif]

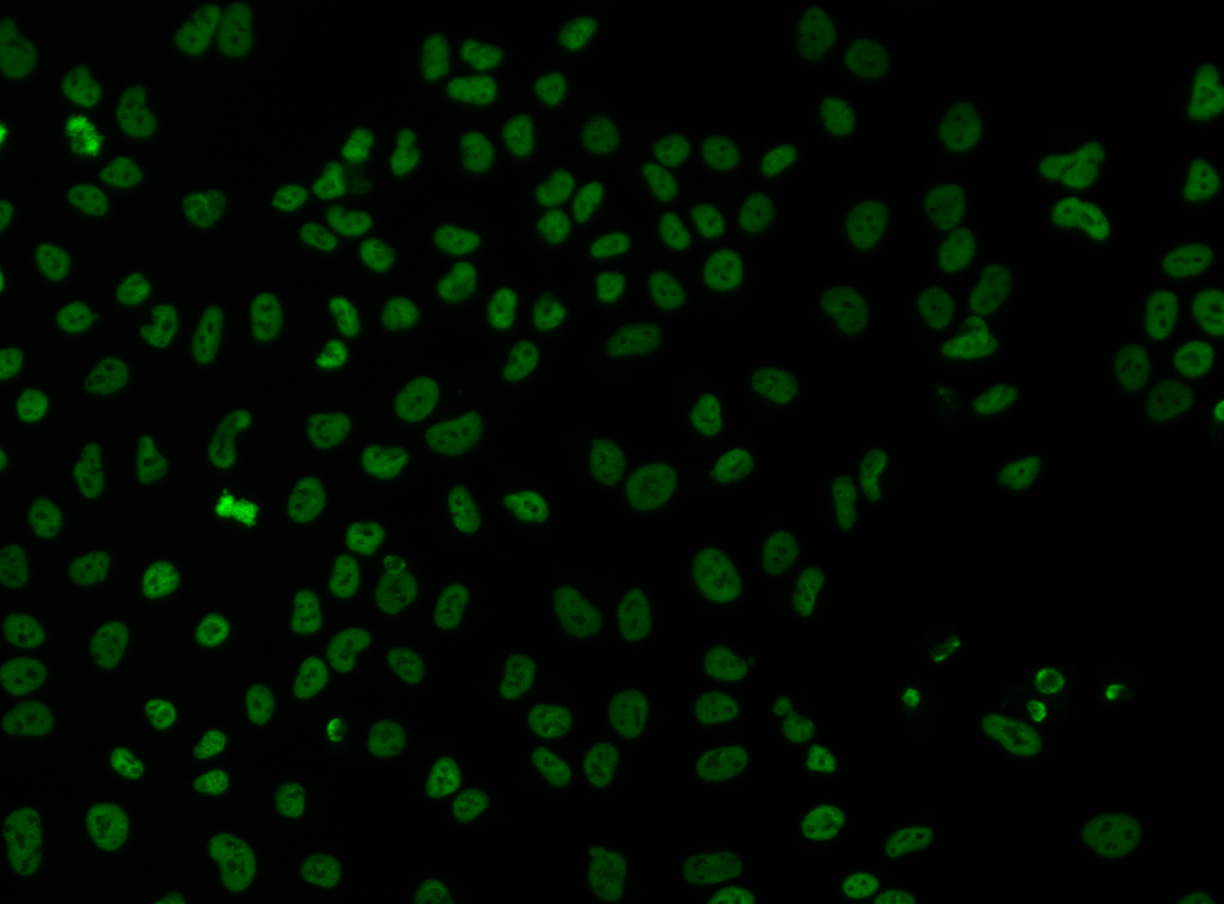

Supplement: Supplementary file 4 — Source Data [file 41467_2024_49347_MOESM4_ESM.zip › Source Data/Figure 4/Fig 4.A_Images Immunofluorescence/Spn live_1┬░/H3K4me2_GFP.tif]

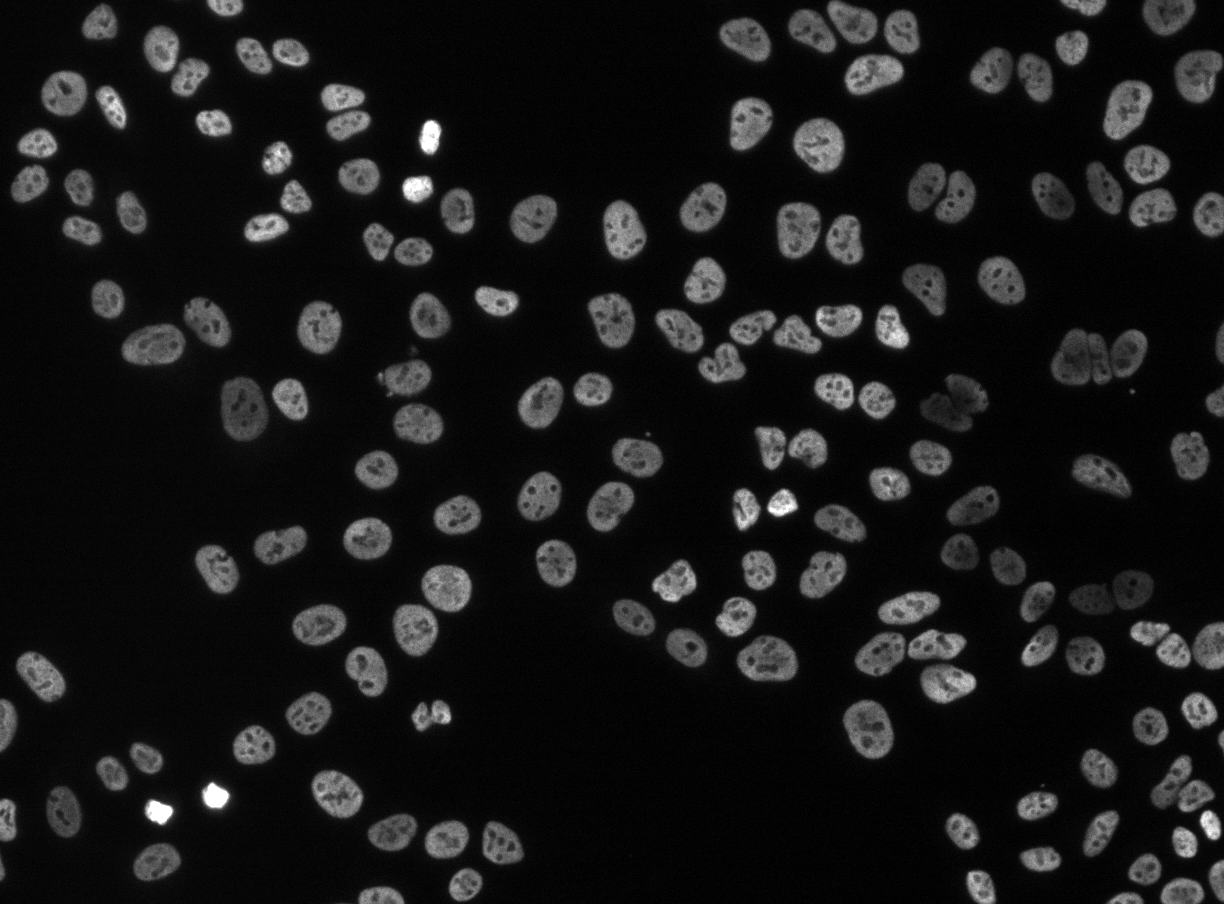

Supplement: Supplementary file 4 — Source Data [file 41467_2024_49347_MOESM4_ESM.zip › Source Data/Figure 4/Fig 4.A_Images Immunofluorescence/Spn inactived_PI/A1_01_2_5_GFP_001.tif]

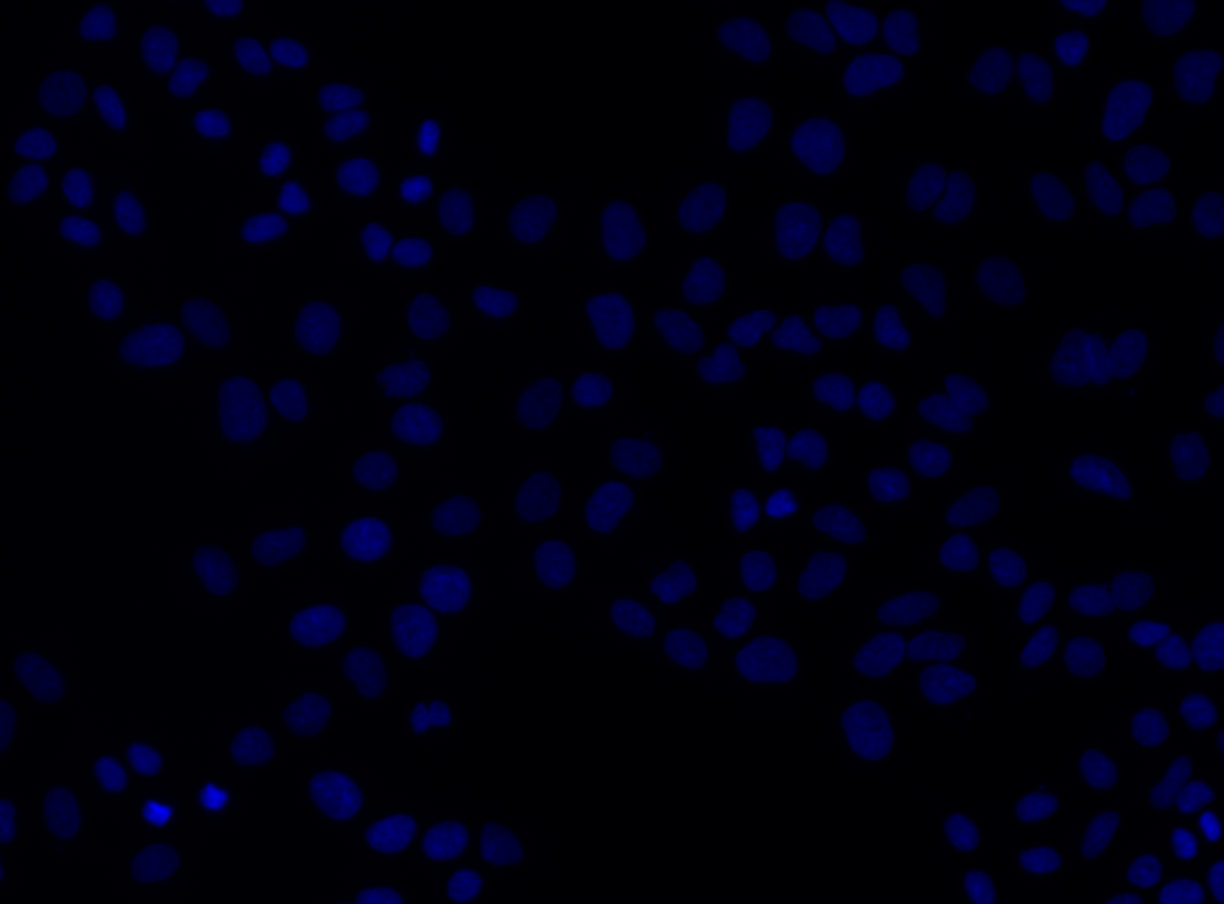

Supplement: Supplementary file 4 — Source Data [file 41467_2024_49347_MOESM4_ESM.zip › Source Data/Figure 4/Fig 4.A_Images Immunofluorescence/Spn inactived_PI/DAPI.tif]

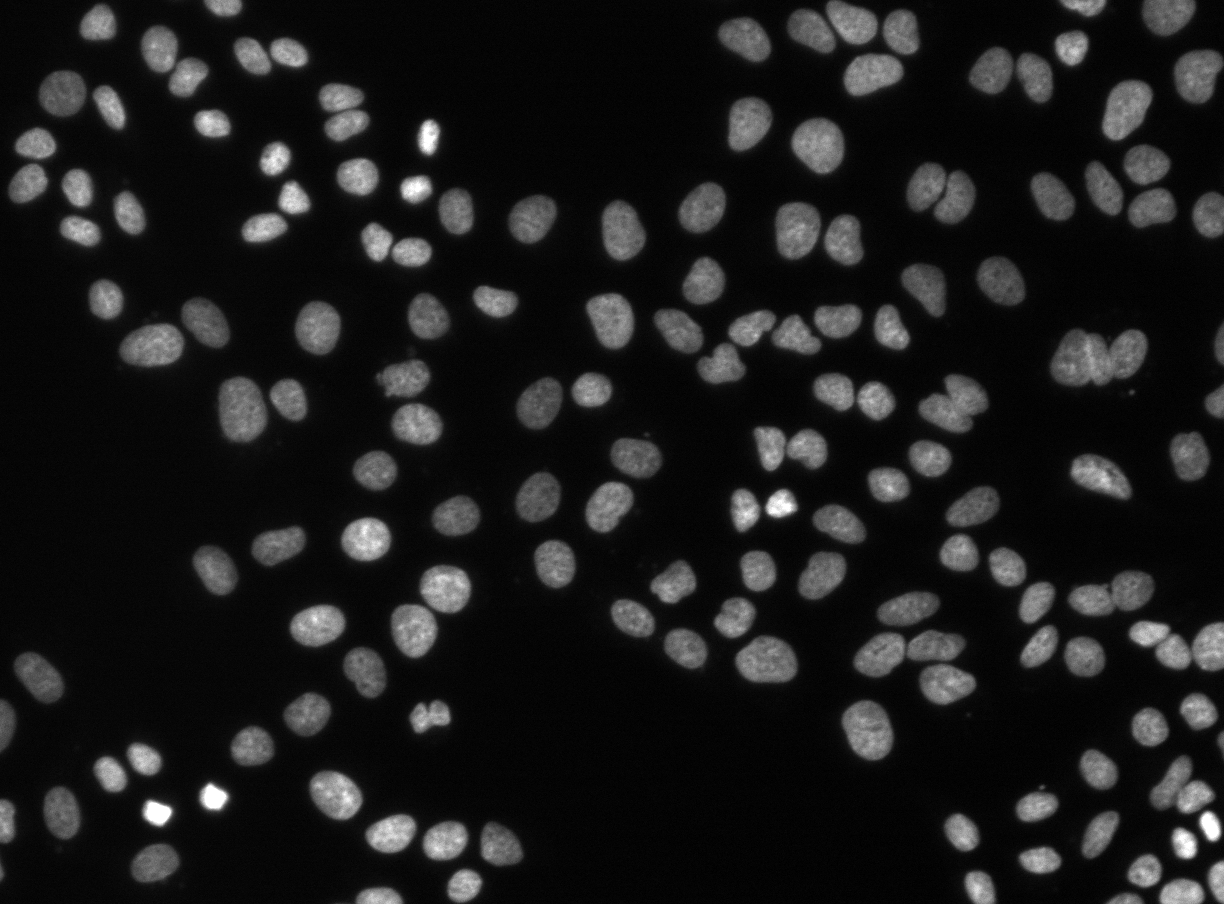

Supplement: Supplementary file 4 — Source Data [file 41467_2024_49347_MOESM4_ESM.zip › Source Data/Figure 4/Fig 4.A_Images Immunofluorescence/Spn inactived_PI/A1_01_1_5_DAPI_001.tif]

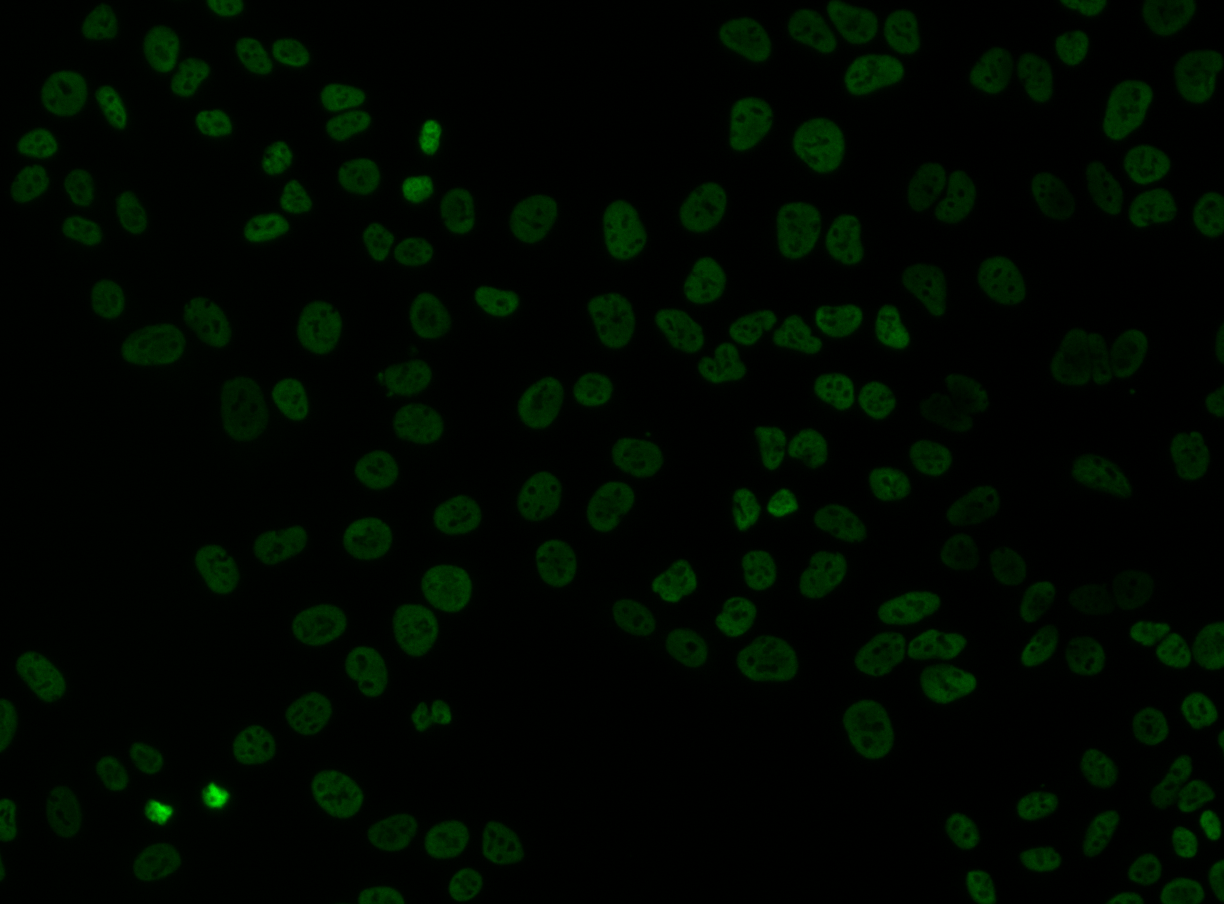

Supplement: Supplementary file 4 — Source Data [file 41467_2024_49347_MOESM4_ESM.zip › Source Data/Figure 4/Fig 4.A_Images Immunofluorescence/Spn inactived_PI/H3K4me2_GFP.tif]
